# A benthic bioindicator reveals distinct land and ocean–Based influences in an urbanized coastal embayment

**DOI:** 10.1371/journal.pone.0205408

**Published:** 2018-10-11

**Authors:** Samantha E. M. Munroe, Jack Coates-Marnane, Michele A. Burford, Brian Fry

**Affiliations:** Australian Rivers Institute, School of the Environment, Griffith University, Nathan, Queensland, Australia; University of Sydney, AUSTRALIA

## Abstract

Biogeochemical maps of coastal regions can be used to identify important influences and inputs that define nearshore environments and biota. Biogeochemical tracers can also track animal movement and their diet, monitor human coastal development, and evaluate the condition of habitats and species. However, the beneficial applications of spatial biogeochemical analysis are hindered by a limited understanding of how tracer distribution is affected by different land and ocean–based influences. To help address these knowledge gaps, we determined the spatial trends of three stable isotopes (δ^13^C-carbon, δ^15^N-nitrogen, δ^34^S-sulfur) and 13 major and trace elements in an urbanized coastal embayment (Moreton Bay, Australia), as incorporated into the muscle tissue of a marine consumer, the eastern king prawn *Melicertus plebejus*. Results were used to identify unique biochemical regions within the bay and to discuss how spatial patterns in tracers could be used to indicate the relative importance of catchment, urban and offshore drivers in coastal bays. Discriminant analysis identified seven biogeochemical regions that were likely distinguished by variation in catchment, urban, and offshore input, and habitat type. δ^13^C and δ^15^N patterns suggested nearshore areas could be distinguished by increased sediment resuspension and higher wastewater inputs from catchments. High inshore lead (Pb) and copper (Cu) concentrations were likely the result of urban input. Arsenic (As) and cadmium (Cd) increased further from shore. This trend implied oceanic influences were a significant control over As and Cd bioavailability. Cobalt (Co) and rare earths were also used to differentiate some nearshore areas, but incongruent distribution patterns in Co suggested it may be less reliable. Overall, results indicated that δ^15^N, δ^13^C, Cd, Cu, Pb and rare earth elements were the most reliable tracers to differentiate nearshore and offshore environments, and catchment–based effects. We encourage future studies to consider using a similar multivariate approach in coastal spatial analysis, and to include unrelated tracers that reflect distinct coastal influences.

## Introduction

Coastal and estuarine ecosystems are diverse, productive environments that contain a wide variety of habitats and species [1–3]. Healthy coastal ecosystems provide critical services for human communities, including waste-water purification, erosion control, and support for coastal fisheries [4]. However, coastal areas are also highly dynamic and often heavily developed environments that experience substantial and sometimes abrupt spatial changes across a range of abiotic and biotic factors, such as offshore water exchange [5], or catchment and urban inputs [6, 7]. The condition of coastal habitats and their capacity to support biodiversity is largely determined by spatial variation in these and other environmental parameters [8, 9]. Consequently, mapping physical, biological, and biogeochemical variations in coastal environments is essential for effective monitoring, management, and conservation.

Coastal zones and communities have traditionally been defined by structural factors such as exposure, tidal range, sediment supply, and climate. Biogeochemistry offers a different perspective on the configuration of coastal regions that incorporates both structural and process-based information. Spatial patterns in biogeochemical tracers, such as stable isotopes or trace metals, can be used to evaluate fine and large-scale abiotic and biotic variation in aquatic ecosystems [10–12]. For example, coastal rivers that drain catchments with unique geological and anthropogenic influences will release distinctive mixtures of sediments, nutrients, and pollutants into coastal basins [12–14]. Biogeochemical maps of these tracers can then be used to identify these influences, define distinct biogeochemical regions, and evaluate the condition of nearshore habitats and species. The information provided by tracers and biogeochemical mapping also enables landscape-level evaluations of changes in coastal communities. For example, changes in tracer concentrations and distribution patterns over time can be used to monitor the effects of coastal development or the success of management programs. Biogeochemical maps therefore allow us to better define input areas and processes within coastal systems.

The beneficial applications of biogeochemical mapping and monitoring programs are limited by our relatively poor understanding of how tracers disperse through coastal habitats and the spatial relationships that may exist between some tracers. As a result, most biogeochemical studies rely on only one or a few tracers (e.g. stable isotopes or trace metals), and there is little work that shows how different tracers are simultaneously transferred through coastal habitats. Using a small number of tracers can also limit the scope and precision of a particular study. For example, coastal ecosystems often have a larger number of sources than isotope tracers, which can lead to inaccurate model outputs [15, 16]. The isotope values of distinct food sources or habitats may also overlap, making it difficult to distinguish different populations or environments using biochemical methods [17–19]. In dynamic and diverse coastal systems, additional and disparate tracers can improve spatial analysis by further distinguishing different habitats, populations, and environmental influences at smaller and more ecologically relevant scales [10, 20]. Therefore, work that clarifies spatial biogeochemical trends in coastal areas, and includes a range of potential tracers, will advance a wide range of research fields and applications.

Bioindicators offer a valuable and practical way to measure biogeochemical variation in coastal environments [21, 22]. A bioindicator is any organism that has the capacity to accumulate unique biogeochemical “fingerprints” that integrate and reflect local tracer concentrations from multiple potential sources (water, sediment, and diet) [20]. These fingerprints not only reflect tracer exposure or availability, but also the local environmental conditions which affect tracer bioavailability and uptake [23, 24]. Secondary generalist consumers may be particularly valuable bioindicators because their diet, and resultant trace element values, represent an integration of disparate biogeochemical sources [25]. For example, lead (Pb) measured in the muscle tissue of the opportunistic consumer crab *Callinectes arcuatus* was used to determine Pb sources in the Gulf of California (Mexico) [26]. Increased metal accumulation in greentail prawns *Metapenaeuss bennettae* closely corresponded to sediment concentrations in contaminated estuaries in New South Wales, Australia [27]. Thus consumer tissue values can provide unique and useful insight into the distribution of bioavailable tracers.

The primary goal of this study was to create exploratory biogeochemical maps of an urbanized, coastal embayment (Moreton Bay, Australia) using stable isotope, major, trace, and rare earth element values as determined in the muscle tissue of the benthic invertebrate species, the eastern king prawn *Melicertus plebejus*. The results were used to investigate different tracers as identifiers of key costal influences. *Melicertus plebejus* was chosen as a potentially useful bioindicator because it has relatively fast isotope turnover (1–2 weeks)[28–30], and are one of the few benthic generalists that can be found in almost all areas of Moreton Bay [31–34]. Although *M*. *plebejus* is mobile, its residency time in Moreton Bay is relatively long in comparison to its tissue turnover rate [35]. As a result, *M*. *plebejus* tracer concentrations should reflect regional trends in bioavailable tracers. Where applicable, we discuss the utility of *M*. *plebejus* as a bio-indicator, and how *M*. *plebejus* tracer accumulation can be used to better understand tracer dispersal. Our research will contribute to a better understanding of tracer dispersal patterns in urbanised bays, how tracers can be used to identify key coastal influences, and increase the number of potential tracers for future applications.

## Materials and methods

### Ethics statement

Specimens were collected in cooperation with the Long-Term Monitoring Program (Fisheries Queensland), which conducts annual fishery-independent recruitment monitoring surveys of *M*. *plebejus* in southeast Queensland. Because samples were collected under this government program, no additional permits were required. No ethics permits were required from the Griffith University Office of Research because the study species was an invertebrate. Specimens were euthanized quickly and humanly by freezing individuals at -20°C in on-board freezers.

### Study site

**Fig 1:**
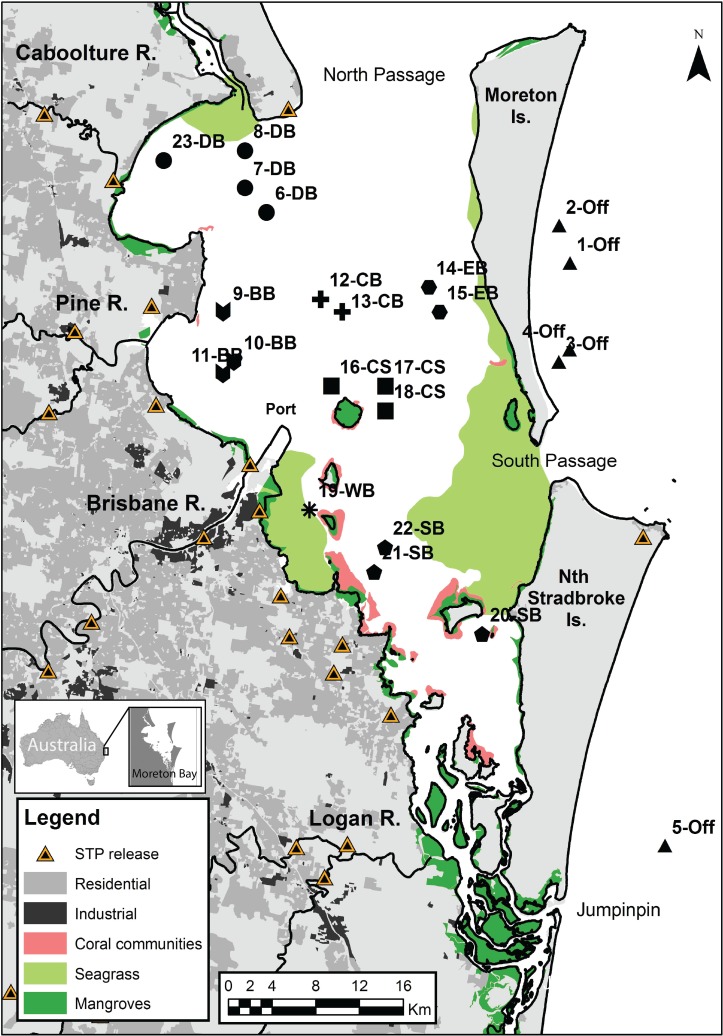
Map of Moreton Bay, Queensland, Australia. Points and numbers indicate sampling sites. Shapes & colours indicate grouped sample areas, Bramble Bay (star, BB), Deception Bay (blue circle, DB), Central Bay (grey circle, CB), Central South (white square, CS), Eastern Bay (red square, EB), Offshore (yellow diamond, Off), South Bay (black triangle, SB), Waterloo Bay (brown inverted triangle, WB). STPs indicate the locations of sewage (i.e. waste water) treatment plant outlets.

Moreton Bay is a large (1845 km^2^), shallow (mean depth = 8m), semi-enclosed estuary located on the southeast coast of subtropical Queensland, Australia (Fig 1). The bay is bounded by two barrier islands (Moreton Island and Stradbroke Island) that limit exchange with oceanic waters. Moreton Bay is adjacent to the city of Brisbane which has a steadily growing current population of approximately 2.25 million people. The surrounding areas are dominated by rural communities and are primarily used for agriculture and peri-urban activities. The bay contains a variety of habitats including rocky and sandy beaches, seagrass, mudflats, mangroves, river deltas, and coral communities. The bay also receives periodic terrestrial run-off from four primary rivers (Caboolture, Pine, Brisbane, Logan). Sewage treatment plant (STP) outlets are located along each of these primary river systems. The Brisbane River is the largest coastal river in Moreton Bay and drains both agricultural areas in the upper catchment as well as the urban centre of Brisbane near the coast. Average flow is low at 5 m^3^s^-1^, and water residence time in the lower river near the coast is high at 50–100 days [36]. Summer dominated rainfall in combination with large storms can lead to increased river flow and periodic flood events that discharge water and associated sediment and contaminants into the bay [37]. The Brisbane River contains the largest and most active industrial port in the bay, however smaller industrial and recreational marinas are found all along the Moreton coastline. Freshwater inputs can eventually be transported out of the bay through the north passage by a tidal current that initially brings oceanic water southward along the eastern margin of the bay, but then turns northward along the western shore. Salinity in the bay ranges from 24.7 to 37.6 g/L (mean = 34.7 ± 1.69) and generally increases with distance from major coastal river mouths [38]. Salinity values are relatively consistent throughout the year but can decrease substantially (< 20 g/L) during periods of increased river flow or flooding. However, no major floods occurred during our sampling period (November-December 2013). Annual water temperature ranges from 13.8 to 30.5°C (22.4± 3.5) [38]. Water temperatures between November and December typically range from 21.9 to 29.2°C (24.8±1.4). Water quality monitoring studies over more than 10 years conclude that the nearshore areas (e.g. Bramble Bay, Deception Bay, Waterloo Bay) have lower water quality scores than areas further from the mainland (Central Bay and Eastern Bay) [39].

### Study species

*Melicertus plebejus* is endemic to eastern Australia and is found from 20°S (central Queensland) to 42°S (north-eastern Tasmania) [40, 41]. Juvenile *M*. *plebejus* use nearshore habitats as nurseries for two to four months in a one to four year life cycle [42]. As adults they eventually migrate offshore and do not return to estuarine waters [43, 44]. Females spawn and release their eggs in oceanic waters. The eggs hatch, and a proportion of the free swimming post-larvae are trapped in tidal advective envelopes, dragging them into coastal estuaries to settle and grow throughout their juvenile phase [45–47]. Moreton Bay is considered an important nursery for *M*. *plebejus* at the northern end of their range [48]. In Moreton Bay prawns recruit from nearshore nursery habitats (which include bare substrate and seagrass habitats), to central areas of the bay in the austral spring, i.e. October and November [35, 48, 49]. *Melicertus plebejus* ultimately migrate offshore, most likely via the northern passage, in January and February. Therefore all individuals collected in this study were juveniles. *Melicertus plebejus* is nocturnal and individuals burrow into the substrate during the day. In general, prawns have the ability to swim, although their primary mode of locomotion is walking. *Melicertus plebejus* is an opportunistic consumer. Its diet consists of small shellfish, other crustaceans, and worms, and it incidentally consumes plant material, detritus, sediment and micro-organisms [31–33].

*Melicertus plebejus* was selected as a bioindicator species because preliminary trawls and collections in Moreton Bay indicated that *M*. *plebejus* is one of the few benthic generalist consumers that can be found throughout Moreton Bay in a wide range of habitats [34, 48]. Thus, by using *M*. *plebejus* as a study species, it was possible to examine tracer distribution across the majority of the bay without having to compare species with different tracer integration and accumulation patterns. Although *M*. *plebejus* is a mobile species, its migration patterns within Moreton Bay are well-understood and uni-directional (i.e. prawns do not exit and re-enter nearshore areas)[35]. Moreover, isotopic turnover of juvenile *M*. *plebejus* is relatively fast. Juveniles grow rapidly within nearshore habitats, increasing approximately 1000 times in biomass before they migrate offshore [45, 50]. After 1–2 weeks of consuming a new food source, juvenile muscle tissue should reach full isotopic equilibrium [28–30]. Finally, previous research has shown prawns can be valuable bio-indicators of regional trends in anthropogenic pollution [27, 51]. Therefore, although *M*. *plebejus* movement had the potential to affect our interpretations of tracer distribution, it was expected that overall *M*. *plebejus* tissue values would be effective indicators of local biogeochemical conditions and trends.

### Sample collection and analysis

Prawns were collected from eight areas in Moreton Bay in cooperation with the Long-Term Monitoring Program (Fisheries Queensland), which conducts annual fishery-independent recruitment monitoring surveys of *M*. *plebejus* in southeast Queensland. These areas were selected because they were thought to have distinct terrestrial and offshore inputs, and they contain different habitat types. We predicted these eight areas would exhibit distinct biogeochemical patterns which could be used to distinguish each area and identify different potential coastal influences. These eight areas included four inshore areas; (1) Deception Bay, which receives terrestrial input from the Caboolture River and northward flow from the Brisbane River; (2) Bramble Bay, which receives input from the Brisbane River and Pine River; (3) Waterloo Bay, which receives relatively limited input from the Brisbane River (as compared to Bramble Bay); and the (4) Southern Bay, which receives input from the Logan river, and oceanic water input via a small passage between the barrier islands (Jumpinpin). Bramble Bay primarily contains sandy bottom and mudflat habitats. Deception Bay and Waterloo Bay contain both seagrass beds and sandy/ mudflat benthic habitats. The Southern Bay primarily contains sandy habitats, coral, and seagrass. Samples were also collected from the: (5) Central Bay; (6) Eastern Bay; the transition zone between the Southern Bay and Central Bay, which was referred to as (7) Central South; and finally (8) Offshore sites, which were expected to be relatively pristine compared to inshore areas.

At each site, ten prawns representing the range of size classes present were collected using a beam trawl aboard the FRV *Tom Marshal* trawler. This occurred from November to December 2013. Prawns were kept frozen (– 20º C) until they were processed for analysis. Prawns were rinsed with deionized H_2_O, blotted dry, weighed (g) and measured (total length, mm). Tail muscle tissue was dissected and the gut was removed. Muscle tissue was chosen for tracer analysis because muscle tracer values in crustaceans provide conservative estimates of trace metal accumulation [52]. Muscle tissue was expected to provide a more holistic representation of tracer distribution as it can be more strongly associated with diet (i.e. particulate sources)[53, 54] as compared to the gills or hepatopancreas, where values may be more closely associated with tracer availability in the water column (dissolved sources)[54, 55]. The isolated muscle tissue was rinsed three times to remove residual salts. Sub-samples were divided for isotope analysis and major and trace element analysis. Isotope values were determined for each individual, while 10 individuals were combined in composite samples to ensure there was enough sample for trace element analysis.

#### Stable isotope analysis

Isotope samples were oven dried at 60º C for 48 hours and ground into a fine powder using a stainless steel ball mill grinder. 9–10 mg of dried, ground tissue was weighed in tin capsules and an oxidizing agent (vanadium pentoxide) was added to aid combustion of S for δ^34^S analysis. Samples were analysed using a Sercon Europa EA-GSL elemental analyzer and a Hydra-20-20 continuous flow isotope ratio mass spectrometer. Stable isotope ratios were expressed in δ notation as deviations from standards in per mil (‰): δX = [(*R*_sample_/*R*_standard_)-1] x 1000, where X is ^13^C, ^15^N, or ^34^S, *R*_sample_ is the ratio (^13^C /^12^C, ^15^N/^14^N, or ^34^S/^35^S) in the sample, and *R*_standard_ is the ratio in the standard. Laboratory and National Institute of Standards and Technology (NIST) standards were analysed to estimate analytical precision and accuracy. Reference materials were Vienna Pee Dee Belemnite carbonate (VPDB) for δ^13^C, atmospheric N_2_ for δ^15^N, and Vienna Cañon Diablo troilite (VCDT) for δ^34^S. The analytical precision (standard deviation) for NIST standard NBS 127 (n = 6) and NBS 1577 (bovine liver, n = 6) was 0.1‰ and 0.1‰ for δ^13^C, 0.1‰ and 0.3‰ for δ^15^N, and 0.6‰ and 0.7‰ for δ^34^S. Duplicate samples were run as an additional measure of analytical precision. The analytical precision for duplicates was calculated as the mean and standard deviation of the absolute difference between each set of duplicate samples. Differences were 0.2 ± 0.11‰, 0.3 ± 0.26 ‰, and 0.3 ± 0.34‰ for δ^13^C, δ^15^N, and ^34^S, respectively.

#### Major and trace element analysis

Composite samples for trace element analyses were freeze-dried for 48 hours and powdered using a zirconium shatter box grinder. Samples were prepared using microwave-assisted digestion with laboratory grade distilled nitric acid (HNO_3_) (70%), and analysed using an Agilent 7700 Series inductively coupled plasma mass spectrometer (ICP-MS). The primary certified reference materials (CRMs) included NIST 2976 (muscle tissue for trace elements), AGAL 3 (prawn tissue for trace elements), BCR 668 (muscle tissue for rare earth elements), and NCSZ 73034 (prawn tissue for trace metals and rare earths). A secondary reference material (SRM) was developed using locally sourced prawns, with means and standard deviations established with reference to CRM results of the same analytical run. The practical quantification limit (PQL) for individual elements were established by running 10 digest blanks with PQL calculated as: PQL = 3 × (mean + (3 standard deviations)). Spiked recovery tests were carried out to optimize the ICP-MS settings for analytes. Major (Al, Fe, Mn), trace (As, Cd, Co, Cu, Ni, Pb, V, Y), and rare earth (Ce, La) elements were simultaneously measured. Duplicate samples were run as an additional measure of analytical precision. The analytical precision for duplicates was evaluated by calculating the coefficient of variation, (100 x (standard deviation/mean)) for each set of duplicate samples (S1 Table).

### Data analysis

#### Spatial interpolation

Spatial variation in prawn stable isotope, and major and trace elements values between sites were initially visualized using circle plots in ARC GIS. In addition, an interpolated raster layer was generated for each variable using the minimum curvature spline technique with barriers; whereby the interpolated surface was constrained by the geographic features of the bay (i.e. Moreton and Stradbroke Islands). The resultant isoscapes, and what will be referred to as “tracerscapes”, were used to discuss broad trends in tracer dispersal across Moreton Bay.

#### Spatial isotope analysis

All remaining data analysis was done in the R environment version 3.4.4. ANOVA and posthoc Tukey’s HSD tests were used to compare prawn isotope values between each sample area. Standard elliptical areas corrected for small sample sizes (SEA_c_) were calculated for each sample area and used to estimate isotopic niche overlap between groups [56]. Each SEA_c_ encompassed 40% of the data in each sample area. Corrected ellipses were fitted with maximum likelihood estimations. Overlap between each SEA_c_ was measured as a percent (%). Standard elliptical area and overlap were calculated using the package *SIAR* [57] and *SIBER* [56]. SEA analysis was used in conjunction with the ANOVA and Tukey’s HSD tests to identify isotopically distinct regions of Moreton Bay.

#### Discriminant analysis

Linear discriminant analysis was used to isolate the isotopes and elements (together referred to as tracers) that could distinguish the eight sampling areas and correctly assign prawn composites to known sample areas. Prior to linear discriminant analysis (LDA), Z-scores were calculated for each tracer at each site within each sample area. Z-scores were calculated by subtracting the mean *M*. *plebejus* Moreton Bay value for each tracer from the sample site value, and dividing the output by the standard deviation of the mean [51] ($Z=(X_{i}-\bar{X})/\sigma_{\bar{X}}$). This approach converts all values to a similar standard deviation scale and creates more reliable comparisons between tracers. Mean, standard deviations, and Z-scores of measured values for isotopes for each sample site are listed in S2 Table. Measured and Z-score values for major and trace elements are listed in S3 and S4 Tables, respectively.

Tracers were removed from the LDA model using a step-wise subtraction process that eliminated tracers that exhibited high collinearity or low importance in sample area discrimination. Collinearity was assessed using the tolerance statistic (1-R^2^, where R^2^ is produced from a regression of one predictor variable against the remaining predictors). Tolerance values ranged from 0 to 1, where values close to 0 indicated variables were strongly correlated. The relative importance of a tracer in discriminating between sites was assessed using an F-to-remove statistic. Tracers with small F-to-remove statistics were deemed the least useful in discriminating between sites and were removed from the analysis. “Leave one out” cross validation was used to calculate assignment error rates and assess the accuracy of each subsequent version of the model. LDA models were fitted and assessed using the *klaR* [58] package. The results of LDA were used to identify important biogeochemical regions and tracers in Moreton Bay.

## Results

A total of 249 prawns were collected from 25 sites inside (20) and outside (5) Moreton Bay. Only nine prawns were collected from Site 16-CS. Prawn length ranged from 47–156 mm (mean = 90.7 mm), and wet mass ranged from 0.48–25.6 g (mean = 5.7 g). There were significant differences in prawn length between areas (F_(7, 251)_ = 12.43, *P* < 0.05; Table 1). Prawns from Deception Bay and the Southern Bay were significantly smaller compared to all other areas. Prawns from Waterloo Bay were also smaller than other sites, but were only significantly smaller than prawns collected from the Eastern Bay. The largest individuals were caught Offshore and in the Eastern Bay. Overall, prawns increased in size with distance from the mainland. This pattern was expected as western areas of Moreton Bay are key nursery grounds for *M*. *plebejus*, and as prawns grow, most move offshore through the northern passage of the bay [43, 44].

**Table 1:** Mean (± standard deviation) *M*. *plebejus* size (total length, mm) from eight proposed biogeochemical regions in Moreton Bay.

Area	Mean (± SD)	Tukey’s posthoc test
**Bramble Bay**	93.4 (18.6)	bc
**Central Bay**	97.9(15.6)	bc
**Central South**	92.8 (18.6)	bc
**Deception Bay**	74.3(16.6)	a
**Eastern Bay**	108.5 (16.5)	b
**Offshore**	100.2 (21.2)	bc
**Southern Bay**	75.3 (14.2)	a
**Waterloo Bay**	88.3 (13.9)	b

Letters indicate results of Tukey’s posthoc test. Different letters denote significant differences in *M*. *plebejus* size between regions (*p* < 0.05)

### Spatial isotope analysis

**Fig 2:**
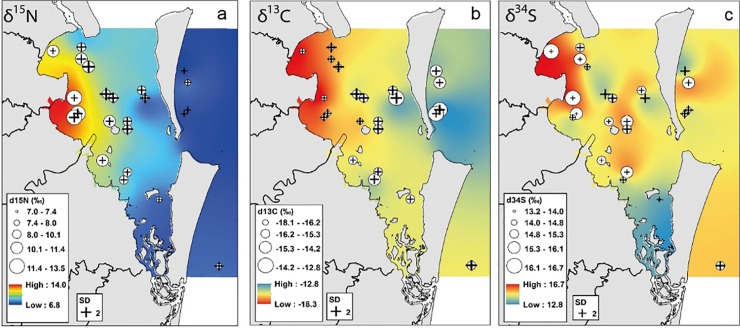
Spatial interpolation results and circle plots of stable isotopes values in *M*. *plebejus* muscle tissue from Moreton Bay. (a) δ^15^N, (b) δ^13^C, and (c) δ^34^S.

δ^13^C and δ^15^N analyses consistently demonstrated that prawn isotope values varied significantly between coastal areas. Circle plots and isoscapes indicated that δ^15^N values gradually decreased with increased distance from shore, while δ^13^C gradually increased (Fig 2A and 2B). In contrast, δ^34^S values were highly variable between sites with no consistent offshore trend (Fig 2C), although mean prawn values were highest in Deception Bay. Offshore areas had significantly lower δ^15^N (F_(1, 247)_ = 75.67, *P* < 0.05) and significantly higher δ^13^C than inshore areas (F_(1, 247)_ = 36.59, *P* < 0.05). There was no significant difference in δ^34^S between inshore and offshore sites (F_(1, 247)_ = 1.11, *P* > 0.05).

**Fig 3:**
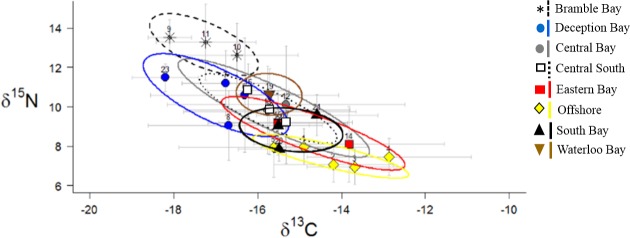
δ^13^C and δ^15^N biplot of *M*. *plebejus* muscle tissue from eight proposed biogeochemical regions in Moreton Bay. Points are the mean values for each site (numbers correspond to site numbers). Grey bars are standard deviations. Ellipses are 40% SEAc.

There were also significant differences in prawn δ^13^C (F_(7, 241)_ = 13.79, *P* < 0.05) and δ^15^N (F_(7, 241)_ = 35.67, *P* < 0.05) between sample areas within Moreton Bay (Table 2). There were no significant differences in prawn δ^34^S between any sample areas (F_(7, 241)_ = 1.65, *P* > 0.05). Prawns from nearshore areas, i.e. Bramble Bay, Deception Bay, and Waterloo Bay, generally had higher δ^15^N and lower δ^13^C compared to most other areas. Bramble Bay prawns had significantly higher δ^15^N values compared to all other areas (Table 2; Fig 3). Bramble Bay prawns also had significantly lower δ^13^C values than all other areas with the exception of Deception Bay, and exhibited low isotopic overlap with all sample areas (0–10%). Waterloo Bay and Deception Bay prawns exhibited moderate isotopic overlap (13–41%), where prawns from Deception Bay had lower δ^13^C and higher δ^15^N than prawns from Waterloo Bay. However values were not significantly different between these two bays.

**Table 2:** Mean (± standard deviation) of δ^15^N and δ^13^C values in *M*. *plebejus* muscle tissue from eight proposed biogeochemical regions in Moreton Bay.

Area	Mean δ^13^C (± SD)	Tukey’s results	Mean δ^15^N (± SD)	Tukey’s results
**Bramble Bay**	-17.2 (1.2)	a	13.1 (1.5)	a
**Central Bay**	-15.6 (2.2)	d	9.9 (2.4)	dce
**Central South**	-15.7 (1.6)	cde	9.9 (1.7)	dce
**Deception Bay**	-16.9 (1.7)	acf	10.5 (2.0)	c
**Eastern Bay**	-14.6 (2.1)	bde	8.6 (1.8)	bd
**Offshore**	-14.2 (1.8)	b	7.4 (1.1)	b
**Southern Bay**	-15.2 (1.2)	bde	8.7 (1.0)	d
**Waterloo Bay**	-15.7 (0.7)	def	10.6 (1.0)	c

Letters indicate results of Tukey’s posthoc test (Tukey’s results). Different letters denote significant differences in *M*. *plebejus* δ^13^C and δ^15^N between regions (*p* < 0.05)

Central Bay and Central South prawns had mean δ^13^C and δ^15^N values that transitioned between nearshore and offshore sites, but individual prawns from these areas exhibited a wide range of δ^13^C and δ ^15^N values. Central Bay and Central South prawns also exhibited a nearly identical range of isotope values (58–100% overlap). Central Bay and Central South prawns also exhibited high isotopic overlap with all other groups except for Bramble Bay and Offshore prawns (23–78%). Central and Central South prawn isotope values were significantly different from Bramble Bay and Offshore prawns. Central Bay prawns also had significantly higher δ^13^C than prawns collected from Deception Bay.

**Fig 4:**
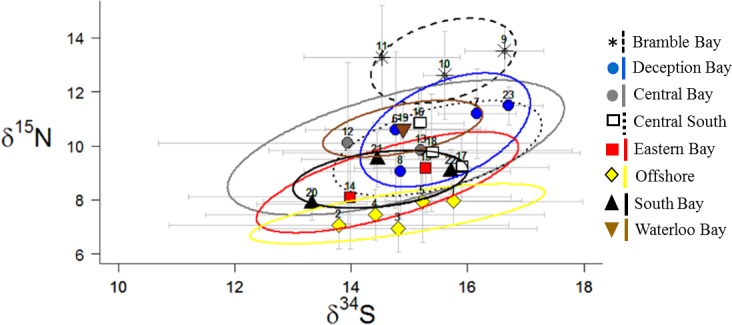
δ^34^S and δ^15^N biplot of *M*. *plebejus* muscle tissue from eight proposed biogeochemical regions in Moreton Bay. Points are the mean values for each site (numbers correspond to site numbers). Grey bars are standard deviations. Ellipses are 40% SEAc.

The Eastern Bay, Southern Bay, and Offshore sites had significantly lower δ ^15^N and higher δ^13^C compared to nearshore sites and exhibited low isotopic overlap with nearshore areas (0–32%). Thus Offshore, Eastern Bay and the Southern Bay sites could be distinguished from nearshore areas using δ^13^C and δ ^15^N alone. However, these three areas exhibited moderate to high isotopic overlap (24–79%) and were not significantly different. Eastern Bay and Southern prawns were also not significantly different from Central Bay or Central South prawns. δ^15^N and δ^34^S analysis indicated δ^34^S values were highly variable between sites within the same sample area, and δ^34^S poorly distinguished different areas of Moreton Bay (Fig 4). Overall, isotope analysis indicated there were three broad biochemical regions within Moreton Bay: (1) the three nearshore areas; (2) the two central areas; and (3) Eastern Bay, Southern Bay, and Offshore areas.

### Major and trace element analysis

**Fig 5:**
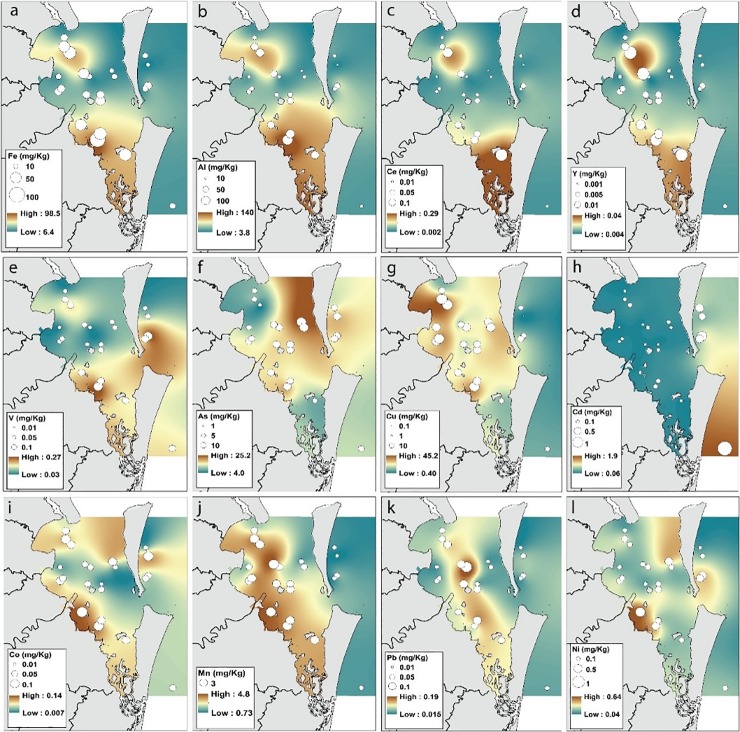
Spatial interpolation results and circle plots of trace elements concentrations in *M*. *plebejus* muscle tissue. Elements are (a) Fe, (b) Al, (c) Ce, (d) Y, (e) V, (f) As, (g) Cu, (h) Cd, (i) Co, (j) Mn, (k) Pb, and (l) Ni.

The elements Al, Fe, Mn, Pb, Cu, Ce, La, and Y had higher concentrations inside Moreton Bay compared to offshore sites, based on circle plots and tracerscapes (Fig 5). Manganese was generally elevated in all nearshore sites. Major elements Fe and Al, rare earth elements La, Ce, and the trace element Y (together referred to as Rare Earths plus Yttrium, REY), had correlated distribution patterns. Southern Bay and Deception Bay prawns had the highest concentrations of each of these elements. Prawn Pb values were highest in the Central and Southern Bay. Cu concentrations were highest in nearshore areas and decreased with distance from shore. Co, Ni, and V concentrations were also high within the bay, most notably in Waterloo Bay, however these trace elements also had high concentrations at some offshore sites. In contrast to most other elements, prawn Cd concentrations were higher in offshore than inshore areas. Cd concentrations were low and consistent in inshore areas, while offshore prawn Cd concentrations were high. Prawn As values also increased further from the mainland and were highest in prawns collected from the Eastern Bay and Offshore. However prawns collected from Bramble Bay, Waterloo Bay, and Site 21-SB within the Southern Bay area also had moderately high As values.

### Linear discriminant analysis

**Fig 6:**
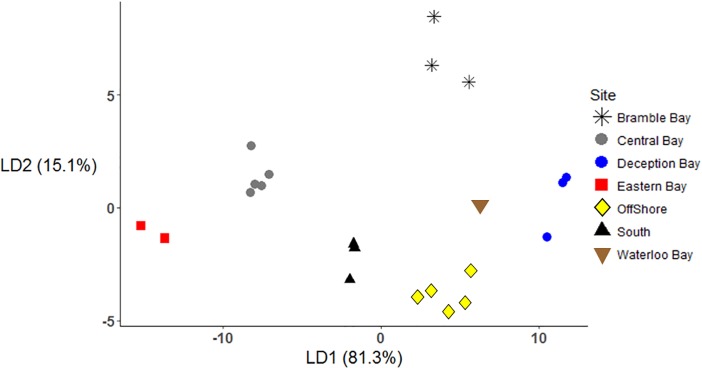
Linear discriminant analysis of stable isotopes, major, and trace element concentrations determined in *M*. *plebejus* muscle tissue collected in Moreton Bay. Samples were grouped into seven proposed biogeochemical regions.

Based on initial LDA, no combination of tracers could distinguish composites from Central and Central South areas. Central and Central South areas were therefore combined into one larger area (hereafter referred to as Central Bay). Initial analysis revealed strong correlations between Al, Fe, Ce, La, and Y. To prevent autocorrelation in the LDA, all but Ce were removed from the analysis. F-to-remove statistics indicated δ ^34^S reduced the discriminatory power of the model and was removed. The subsequent removal of Ni, V, Cd, Mn, and Cu produced the simplest and most well-resolved model with 100% assignment accuracy (Fig 6). Arsenic, δ^13^C, Ce, and Co were the most important tracers in LD1, while δ^15^N was the most important tracer in LD2 (Table 3). Lead made smaller contributions to each LD function.

**Table 3:** Results of Linear discriminant analysis of *M*. *plebejus* muscle tissue from seven proposed biogeochemical regions in Moreton Bay.

Tracer	LD1 (81.2%)	LD2 (15.1%)	LD3 (2.3%)
**δ**^**15**^**N**	1.85	3.233	-0.26
**δ**^**13**^**C**	5.05	-0.419	-0.69
**As**	-9.94	0.36	0.27
**Ce**	-4.45	0.26	1.06
**Co**	4.79	-1.13	0.25
**Pb**	-1.71	-0.12	0.74

Values in brackets are proportion of trace (between-class variance that is explained by successive discriminant functions). Only Linear discriminate (LD) functions with ≥ 1% proportion of trace are included.

Using combined results from each analysis, we identified seven distinct biochemical regions within Moreton Bay. Each area was defined by a unique “fingerprint” or combination of elements: (1) Bramble Bay composites were defined by the highest δ^15^N, low δ^13^C, and high Cu values; (2) Deception Bay composites had low δ^13^C, high δ^15^N, high Cu, low As, and distinctly high levels of REY; (3) Central Bay composites were defined by increased δ^13^C, decreased δ^15^N, high Pb, but relatively lower levels of other trace metals; (4) Eastern Bay composites had low δ^15^N, high δ^13^C, high As, and low levels of most other trace metals; (5) Offshore composites were distinguished by the lowest δ^15^N, high δ^13^C, high Cd, and relatively high As compared to some other areas; (6) the Southern Bay composites had high δ^13^C, low δ^15^N, relatively high Pb, the highest levels of REY, and low As and Cd; finally, (7) Waterloo Bay composites had intermediate δ^15^N and δ^13^C values compared to nearshore and offshore sites, but was primarily defined by high levels specific trace elements, most notably Co.

The inconsistent or patchy distribution of Ni, V, Co and As suggested these tracers may not be as reliable as tracers that exhibited more consistent and continuous distribution patterns, or those that were correlated with available sediment data. Therefore, additional LDA models were developed where Ni, V, Co and As were pre-emptively removed in a step-wise fashion and the remaining tracers were used to construct a more conservative assessment of potential biogeochemical regions. This approach helped to ensure that the supervised LDA technique was as objective as possible.

The pre-emptive exclusion of V and Ni had no effect on the final LDA model or model accuracy. This was not surprising as the original LDA indicated these tracers had low F-to-remove values. However, the subsequent removal of Co changed the LD functions and reduced assignment accuracy to 85% (Table 4). Without Co, the LDA model could not distinguish Central Bay and Waterloo Bay composites. The subsequent removal of As resulted in another unique set of LD functions (Table 5), but maintained 85% assignment accuracy. However, without As it was more difficult to distinguish composites from Deception Bay and Southern sites. This would suggest that the removal of Co and As had a substantial effect on overall LDA accuracy. When Ni, V, Co, and As were excluded from analysis, the final LDA model indicated there were five to six conservative bioregions in Moreton Bay; (1) Bramble Bay, (2) Eastern Bay (3) Offshore sites, (4) Central Bay and Waterloo Bay, which could not be distinguished, (5) the Southern Bay and (6) Deception Bay, however the two latter regions were more difficult to distinguish compared to the original LDA model. Comparisons between different LDA models indicates that while Co and As were important for complete and accurate site discrimination, models that only included the more reliable tracers were still able to differentiate the most prominent biogeochemical regions in Moreton Bay.

**Table 4:** Results of linear discriminant analysis of *M*. *plebejus* muscle tissue from seven proposed biogeochemical regions in Moreton Bay with Ni, V, and Co pre-emptively removed prior to analysis.

Tracer	LD1 (61.8%)	LD2 (28.3%)	LD3 (5.8%)	LD4 (3.0%)
**δ**^**15**^**N**	-1.95	4.48	1.51	-0.72
**δ**^**13**^**C**	0.32	3.31	0.05	-0.67
**As**	2.00	-2.12	1.57	0.0
**Cd**	1.65	1.01	-0.07	-0.24
**Ce**	0.85	-1.14	0.52	0.38
**Cu**	-0.18	-1.41	-0.22	1.46
**Mn**	-0.89	-1.50	-0.60	-1.52

Values in brackets are proportion of trace (between-class variance that is explained by successive discriminant functions). Only Linear discriminate (LD) functions with ≥ 1% proportion of trace are included.

**Table 5:** Results of linear discriminant analysis of *M*. *plebejus* muscle tissue from seven proposed biogeochemical regions in Moreton Bay with Ni, V, Co, and As pre-emptively removed prior to analysis.

Tracer	LD1 (74.1%)	LD2 (20.2%)	LD3 (4.4%)	LD4 (1.0%)
**δ**^**15**^**N**	0.73	-3.97	-1.05	0.59
**δ**^**13**^**C**	2.23	-1.55	-0.79	1.05
**Cd**	1.8	-0.55	-0.40	-1.39
**Cu**	-0.87	0.86	1.46	-0.16
**Mn**	-1.54	1.28	-1.25	-0.33

Values in brackets are proportion of trace (between-class variance that is explained by successive discriminant functions). Only Linear discriminate (LD) functions with ≥ 1% proportion of trace are included.

## Discussion

Our study identified significant spatial variation in a range of tracers in a dynamic, urban coastal environment. Most tracers displayed a gradual but clear west to east trend away from the coast, and the strongest biochemical distinction was between prawns collected from more oceanic (Offshore and Eastern Bay) and nearshore environments. Almost all of the tracers in this study could be used to monitor this transition. Based on these results, the shift from inshore to offshore environments likely strongly influenced the biochemical values of prawns in Moreton Bay. This trend is consistent with the well-established coastal influences typically used to define nearshore ecosystems. However, inshore areas and embayments were also readily distinguished by variation in trace elements. Stable isotopes were used to identify broader biochemical patterns and a few specific areas (e.g. Bramble Bay), while less commonly used tracers, such as REY, identified smaller biochemical regions. Discriminant analysis also revealed distinct biochemical “fingerprints" that could be used to define each area. Previous assessments of Moreton Bay have divided the bay into four regions, based on water quality: Western Bay, Central Bay, Eastern Bay and Southern Bay [39]. Our study extended past evaluations and produced a similar but slightly more differentiated view of the bay, with three regions along the western shore and two regions in the central bay. For example, Bramble Bay prawns had high levels of potential pollutants such as Cu and δ^15^N (which can indicate high levels of wastewater input [59]), and low δ^13^C (which suggests low levels of seagrass cover [11]). This combination of tracers suggests Bramble Bay is best defined by a “polluted nearshore” fingerprint. In contrast, the low levels of some urban pollutants and high δ^13^C in the Southern Bay prawns suggests this area is best defined as a relatively “pristine seagrass” environment [11]. “Oceanic” prawns were readily identified by a combination of low Cu, Pb, and δ^15^N, but high Cd. Different regions of the bay were ultimately identified using a variety of disparate biochemical tracers that in-turn were indicative of variable land-based or oceanic inputs and local processes. These findings demonstrate that using stable isotopes and trace elements with a spatially explicit, multivariate approach can substantially increase the accuracy and precision of coastal spatial analysis.

The use of *M*. *plebejus* as a bio-indicator was supported by the general consistency between prawn tracer values and abiotic values from previous studies in Moreton Bay. For example, Costanzo et al. [60, 61] found that wastewater treatment plants were the primary source of elevated nitrogen loading and δ^15^N in Moreton Bay. δ^15^N was highest near coastal river mouths and adjacent bays, then decreased with distance from shore. Sediments within Bramble Bay and other nearshore areas also have relatively high δ^15^N content compared with other areas (Coates-Marnane pers. comm. 2016). This is consistent with spatial trends in prawn isotope values. Heavy metals that are often used as indicators of anthropogenic pollution were highest in prawns collected along the western shore and within the bay. Higher levels of these tracers in nearshore prawns is consistent with current water quality assessments and sediment patterns in the bay [37, 39]. The spatial regularity and agreement of δ^13^C, δ^15^N, Cu, Pb, and Cd also suggests these tracers are robust indicators of biochemical patterns in Moreton Bay and that *M*. *plebejus* is an effective bio-indicator at a regional level. However, it is important to note prawn migration from inshore nurseries to central bay areas had the potential to affect tracer analysis. The presence of migrant prawns in the Central Bay sites from biochemically distinct nursery areas may have increased tracer overlap between sample areas resulting in inaccurate tracer values and trends. Preliminary analysis of prawn isotope values indicated that one to three individuals from each of the Central Bay sites were recent migrants from nearshore nurseries. Therefore Central Bay composites included a few migrant individuals. However, given the low number of migrants at each site, composite values are likely accurate representations of local values and bay-wide trends. Using a less mobile species, such as sessile suspension-feeder, would have been one way to eliminate this potential complication; however preliminary assessments of the bay suggested it would have been difficult to collect the same sessile suspension-feeding species from all areas of the bay. Because *M*. *plebejus* feed on plants, algae, animals and are found throughout Moreton Bay we believed *M*. *plebejus* tracer values would be a holistic representation of the different tracer sources found in both the sediment and in the water column and that values could be readily compared between sample sites.

*M*. *plebejus* size may also have affected our benthic biochemical maps of Moreton Bay. Previous studies have indicated crustacean size and growth rate can affect tracer uptake and accumulation, although size-based trends are often inconsistent between environments, tracers, and species [62–64]. Juvenile and sub-adult *M*. *plebejus* growth patterns and condition are relatively consistent between different regions of Moreton Bay (Munroe et al. unpublished data), suggesting that the effect of growth on spatial variation in *M*. *plebejus* tracer uptake are small relative to the effects of location and tracer bioavailability. Size-based variation in diet may also have affected tracer values between prawns from different sample areas [65]. Unfortunately, it is difficult to discriminate between size and location-based effects (i.e. food availability) on diet because *M*. *plebejus* ontogeny and habitat use are tightly linked. There is currently no research available analysing changes in *M*. *plebejus* diet with size or location. Given the broad scale of our analysis, the diverse diet of *M*. *plebejus*, and the similarity in *M*. *plebejus* and sediment tracer value trends throughout the bay, it is more likely prawn tracer values were primarily driven by location and bioavailability, rather than *M*. *plebejus* size. Nonetheless, effects of size on *M*. *plebejus* diet is an area of research that requires further study. As is detailed in the following sections, in-depth consideration of each tracer can lead to a better understanding of the potential effects of *M*. *plebejus* as a bio-indicator, the unique environmental influences that define each region, and help determine how different tracers can be applied to future coastal research.

### Stable isotopes

SEA_c_ overlap and discriminant analysis demonstrated δ^13^C and δ^15^N were highly effective and significant tracers. Spatial patterns and biochemical groups derived using δ^13^C and δ^15^N were likely the most reliable because isotope integration is relatively conservative and predicable, and values were measured at the individual level. The most distinctive isotopic pattern was the gradual change in δ^13^C and δ^15^N from the coastline to the offshore area. Relatively low δ^13^C in nearshore areas is often attributed to terrestrial organic and dissolved inorganic carbon (DIC) input into estuaries and coastal basins from coastal rivers. This material is more depleted in ^13^C than marine or offshore sources [17], and can be indirectly incorporated into the diet of local consumers [66, 67]. However, the Brisbane River, which is the largest coastal river in the bay, has low flow rates and high water residency time [36], suggesting that freshwater input from coastal rivers is typically not large enough to inject high volumes of terrestrial organic matter throughout individual bays. Therefore, with the exception of large flood events, direct terrestrial organic input is likely not the primary cause of the depleted ^13^C that was observed in nearshore prawns. However, respiration in marine sediment by bacteria also produces depleted DIC. When shallow water sediment is disturbed and resuspended, the depleted DIC is released from the sediment and re-enters nearshore food chains, thus indirectly lowering the δ^13^C values of nearshore prawns [17].

Prawn δ^15^N values also helped identify areas with high anthropogenic input. Natural terrestrial sources of nitrogen, such as soil, typically have lower δ^15^N than human sources, such as treated wastewater from sewage [17, 60]. As a result, δ^15^N is considered an extremely reliable metric with which to measure wastewater input in coastal environments [59]. Prawns with the highest δ^15^N values were collected near coastal river mouths and within adjacent bays. Prawn δ^15^N then decreased with increasing distance from shore. This pattern strongly suggests that the high δ^15^N in nearshore prawns was the result of wastewater input near the mouths of multiple coastal rivers. This is consistent with previous studies that showed elevated δ^15^N in the water column was due to wastewater input into Moreton Bay [60, 61]. Prawn δ^15^N and δ^13^C may have also been influenced by seagrass habitat distribution. Low δ^15^N and high δ^13^C values are often associated with seagrass habitats [11]. The Southern Bay has the largest and heathiest seagrass beds in Moreton Bay [68]. Southern Bay and Eastern Bay prawns had higher δ^13^C and lower δ^15^N values compared to other nearshore prawns. This could indicate, similar to previous studies of benthic invertebrates in the area, that seagrass habitats and associated food resources are important in these regions [11, 18].

δ^15^N analysis also indicated that benthic and pelagic habitats may respond differently to wastewater nitrogen input over time. Previous studies have shown that modern upgrades to wastewater treatment plants have substantially reduced the amount of excess nitrogen and δ^15^N in Moreton Bay [61, 69]. However, both prawn and sediment δ^15^N patterns suggest that nearshore benthic habitats still contain wastewater N. There are several explanations for this apparent contradiction. Prawns may still be able to access sewage effluent that was buried before the upgrades were installed [69, 70]. Periodic flooding of major coastal rivers can also resuspend and release contaminated sediment from rivers into adjacent coastal areas [61]. Consistent but low levels of wastewater input into Moreton Bay could also increase benthic δ^15^N values, while having no obvious effect on pelagic values. Whereas pelagic environments will quickly process and flush contaminated water out of the bay, contaminated sediment and particulate matter is more likely to settle and persist [71]. It is also possible that elevated nearshore δ^15^N reflects denitrification by benthic bacteria in areas enriched by N inputs from local rivers [72–74]. A combination of these processes, primarily controlled by historical and modern day wastewater input, is the most likely explanation for the observed δ^15^N benthic trend in Moreton Bay.

It was anticipated that δ^34^S would increase model accuracy and provide complementary analytical power [75]. Pelagic and offshore sources, such as seawater, typically have greater δ^34^S values compared to benthic food sources [76, 77]. Therefore, it was expected that *M*. *plebejus* δ^34^S would exhibit a decreasing east-to-west trend, similar to δ^15^N. However, the mean prawn δ^34^S values did not exhibit any consistent pattern, and individual data indicated high within-site variance in all areas of the bay. While it is difficult to explain this result, δ^34^S cycling can be highly complex in estuarine and coastal environments and δ^34^S values can vary substantially between food sources [78]. For example, Oakes and Connolly [79] observed small scale δ ^34^S variation in Moreton Bay seagrasses. If δ^34^S sources are not evenly distributed, this can result in dramatically different consumer δ ^34^S values over a relatively small area. Each prawn trawl could have contained several resource patches with distinct δ^34^S. This may explain the inconsistent *M*. *plebejus* δ^34^S patterns observed in this study.

Ultimately, Moreton Bay isoscapes were able to delineate prawn populations that likely integrated greater amounts of wastewater derived N and nearshore C (Bramble Bay, Deception Bay, Waterloo Bay), populations that likely relied primarily on more offshore marine food sources (Offshore, Eastern Bay), and prawns that were transitioning between these two environments (Central Bay). Although prawns from Southern Bay sites likely incorporated seagrass food resources, isotope analysis could not accurately separate Southern Bay and offshore areas. It is important to note that most geographically distinct sample areas in this study could not be distinguished using stable isotopes. As previously discussed, distinct habitats can have similar isotopic values, which can make it almost impossible to distinguish different sources or environments without additional tracers. If prawns consume isotopically distinct food sources, this can also lead to high individual variability and isotopic overlap [80, 81]. The presence of migrant prawns from isotopically distinct habitats can also increase overlap between distinct areas, although the effect of migration was likely relatively low based on the results of individual isotope analysis. Regardless of the ultimate cause(s), isotopic overlap between sample areas emphasises the importance of a multivariate approach to coastal biochemical mapping.

### Major and trace elements

Major and trace elements, alongside stable isotopes, substantially improved separation between sampling areas. Although trace element distributions were only reported for one season, we had confidence in most tracerscapes because results broadly matched trace element patterns in sediments [37]. The *M*. *plebejus* distribution patterns of several major elements (Al, Mn, and Fe) and trace elements (Cu, Pb, and REY) strongly suggested these elements were derived from terrestrial sources. This is consistent with previous sediment analyses that showed local coastal rivers, most notably the Brisbane River, were the primary entry points for terrestrial soils and trace elements into Moreton Bay [37, 82]. High inshore Mn, Fe and Al concentrations in *M*. *plebejus* likely reflects the higher Mn, Fe and Al found in nearshore, terrestrially-derived silts and clays [37]. Similarly, higher inshore prawn trace element values were likely derived from the higher concentrations of trace elements found in inshore, muddy sediments. Thus, there was broad agreement between prawn and sediment trace element patterns between inshore and offshore sites.

Within the bay, elements exhibited unique inshore distribution patterns. For example, high prawn Pb values were concentrated in the Central and Southern Bay, while high Cu values were dispersed along the mainland coast. This variation was critical to the high resolution of each LDA. The distribution of each tracer can be linked to different sources and processes, including different physiological processing specific to *M*. *plebejus*. Therefore, individual consideration of each tracer can help identify the distinctive chemical and environmental conditions that distinguish different areas of Moreton Bay. Evaluation of trace element distribution is partially limited by a poor understanding of tracer sources and uptake by marine biota. Trace element analysis of individuals would also have increased the analytical power of this study. However, exploratory results in the present study will help identify tracers for individual analysis of coastal bioindicators. In future, trace element distributions in different potential indicators, including sediment and species at different trophic levels, should be combined for a more complete and powerful understanding of element cycling in the bay.

#### Lead

High Pb values helped to distinguish Central and Southern Bay *M*. *plebejus* and was important in the final LDA model. The correspondence between sediment and prawn Pb patterns indicates sediment was likely the primary Pb source for *M*. *plebejus* in Morton Bay, and that *M*. *plebejus* were good bioindicators of benthic Pb distribution. As Pb is a toxic, persistent, immobile element with low solubility, fine sediments are often a significant Pb sink in aquatic habitats [83, 84]. The Brisbane and Logan River are the most significant contributors of anthropogenic, Pb-rich sediment to Moreton Bay [37, 82]. Pb values in both *M*. *plebejus* and sediment in the Central and Southern Bays can likely be attributed to urban run-off and industrial effluent transported via these rivers [26, 37, 82]. However, prawn Pb values from the Southern Bay were higher than expected given the low Pb levels found in Southern Bay sediment. This discrepancy may be due to differences in tracer bioavailability and prawn diet in a seagrass ecosystem as compared to the muddy benthic habitats which dominate other parts of the bay. It is also possible that elevated Pb in Southern Bay *M*. *plebejus* was originally accumulated closer to the mouth of the Logan River. Future fine-scale sampling could resolve these possibilities.

#### Copper

Nearshore prawns (i.e. Bramble, Deception, and Waterloo Bay) had higher Cu concentrations than prawns from all other sample areas. This trend was consistent with previous estimates of Cu values in Moreton Bay sediment [37]; however, elevated Cu was also detected in most inshore prawns. Cu is a common agricultural and industrial pollutant [85–87], and most rivers in Moreton Bay drain catchments with substantial agricultural and urban development. Cu is also a common pollutant near shipyards and marinas as it is a primary component of antifouling paint [88–90]. However, unlike Pb, Cu is a highly mobile, essential element. Elevated Cu in most inshore prawns likely reflects increased Cu exposure from a broad range of more urban and industrial sources in more mobile dissolved and particulate forms. Crustaceans can accumulate and retain more Cu than other biota exposed to similar concentrations of Cu [91]. Therefore, inshore prawns likely also had elevated Cu values because they retained high Cu concentrations as they moved away from more enriched nearshore habitats.

#### Arsenic

Prawn As values increased further from the mainland, and helped separate Eastern Bay composites in the full LD model. This trend is consistent with previous studies that found oceanic prawns in Australia have higher As concentrations than inshore populations (Burford, unpublished data), and prawns imported from other countries [92]. Based on these combined results, there may be a correlation between prawn As and marine habitat exposure in Australian waters. Although some forms of As are toxic [82, 83, 93, 94], As is also a naturally ubiquitous element often found in high concentrations in seawater and marine primary producers, such as phytoplankton and algae [91, 92, 95–98]. Thus, high As in offshore *M*. *plebejus* may indicate increased marine and planktonic food web contributions to its diet. This supposition is supported by similar trends in Moreton Bay prawn δ^13^C. Overall, bulk aquatic animal As values could be used to indicate increased marine or phytoplankton contributions to animal diet, as compared to contributions from estuarine or seagrass habitats which have much lower As values [97, 99, 100]. However, As trends in Moreton Bay were inconsistent between nearshore areas, and will likely fluctuate with seasonal changes in freshwater flow and phytoplankton productivity [95, 98, 101]. Thus, As trends need to be validated with replicate samples over time before it can be included in coastal biogeochemical assessments.

#### Cadmium

Prawn Cd distribution was particularly distinct as inshore prawns had substantially lower concentrations compared to offshore prawns. Although high Cd concentrations in aquatic biota can be associated with human based pollution, our findings suggest elevated prawn Cd was not derived from local anthropogenic sources [83]. High offshore Cd concentrations are consistent with previous studies which have shown north Australian oceanic prawns caught above 30° N have higher Cd concentrations than imported or south Australian prawns [92, 102]. At a national scale, geographic location is the largest determinant of prawn Cd concentrations [102]. However, our results suggest that at a local scale, Cd in northern prawns may be controlled by marine habitat exposure. The mechanism(s) behind this phenomena is not well understood at this time. Potential explanations may include increased bioavailability of Cd in oligotrophic waters, or increased Cd uptake in offshore habitats as a result of the competitive relationship for uptake routes with essential trace elements (Zn, Cu, Mn) [103–106]. Previous work has also indicated that bivalves, mussels and oysters integrate Cd from pelagic (phytoplankton) rather than benthic (sediment) sources [91, 107]. Thus as *M*. *plebejus* transition to an offshore diet (characterized by a greater contribution from pelagic food webs), Cd levels may increase. More research on Cd uptake and distribution is required to better comprehend this local and national trend.

#### Rare earth elements and yttrium

The REYs (Ce, La, and Y), and the correlated major elements Al and Fe, differentiated inshore and offshore sites. REY are typically supplied to the coast through terrestrial run-off from coastal rivers [108, 109] and are consistently higher in freshwater, estuarine, and nearshore environments [110]. However, prawn REY patterns in Moreton Bay were more complex than a simple inshore-offshore trend. Prawns from the Southern Bay and Deception Bay were distinguished from other inshore sites by high REY, Fe, and Al concentrations. Research on REY accumulation in marine animals is limited, but available data indicates that prawn REY concentrations were likely influenced by local differences in tracer availability and environmental conditions (i.e. pH, salinity, redox conditions). REY in freshwater systems are influenced by parent rock concentrations, lithology and weathering, water chemistry, and anthropogenic modification [13, 108, 111, 112]. REY variation between coastal rivers directly influences REY variation between adjacent nearshore habitats [13]. This variation could be passed on to contiguous estuaries and reflected in local benthic species. However, research also indicates that animal REY uptake is nonlinear and can be affected by local conditions, such as changes in pH or alkalinity [110, 113]. Therefore, prawn REY values are not necessarily an accurate representation of abiotic REY distribution.

The correlations between Fe, Al, and REY suggest prawn accumulation of these elements may be related. In marine habitats, REY preferentially bind to Fe-oxyhydroxide colloids found in organic, muddy, claylike sediments, and as a result Fe and REY sediment values are usually correlated [108, 109, 114, 115]. Prawn REY concentrations in Moreton Bay cannot be compared to REY in sediment because sediment REY data are not available for this site. Nonetheless, spatial correlations between prawn Fe, Al, and REY values indicate that prawn REY accumulation may mimic expected correlations in sediment. Similar correlations have been identified in freshwater [116], estuarine [Burford et al. unpublished data] and coastal crustaceans [63]. Our results are also consistent with past work which suggested Fe-oxyhydroxides may be the main forms for REY uptake in amphipods [113]. However, while prawn Al, Fe and REY values had similar distribution patterns, Fe prawn patterns did not correspond with mud and Fe distribution in Moreton Bay sediment, suggesting that REY, Fe, and Al integration is more complex than simple association with muddy sediments and Fe-hydroxides. Despite these unknowns, our results have shown REY elements are useful additions to coastal spatial analysis, and including REY in future studies may prove highly beneficial.

#### Cobalt, nickel, and vanadium

Prawn Co, Ni, and V exhibited incongruent distribution patterns between sample sites, making it difficult to determine the environmental controls that effected tracer distribution. Ni and V were ultimately excluded from all the discriminate models, potentially due to their inconsistent distribution patterns; however Co was important and helped to distinguish Waterloo Bay from Central Bay samples. Although elevated levels of these tracers are usually associated with urban input [117, 118], inconsistent distribution patterns suggest higher concentrations were not exclusively derived from mainland sources. Moreover, elevated prawn Ni concentrations were not associated with elevated Ni concentrations in sediment [37], which suggests prawn Ni was not exclusively controlled by sediment exposure. The biological role of Ni and V in marine animals remains unclear, and the ability of crustaceans to regulate Ni concentrations appears species specific [24, 119–124]. Marine animals can integrate Ni and V from solution [120, 125, 126] and their diet [122, 126, 127], so prawn Ni and V concentrations may be a combination of various sources, resulting in less predictable accumulation and distribution patterns. In contrast, Co is an essential metabolic element [128]. Prawns may accumulate Co to satisfy their metabolic requirements [128, 129]. However, more research on Co uptake and distribution in marine environments is needed before the relevance of Co in this study and its potential applications can be more clearly discussed. Co, V and Ni results should be interpreted with caution because trends were defined by high values at only a few disconnected sites. It is possible these trends were distorted by outliers within the composite sample. Therefore, while discriminant analysis ultimately separated all of the original sample areas, the five to six biogeochemical regions defined by δ^13^C, δ^15^N, REY, Cu, Pb, and Cd, are probably the most robust and reliable groups. Any explanation about Co, Ni, and V distribution should be carefully considered within this context.

## Conclusions

Our study has helped address key knowledge gaps linking tracer dispersal patterns and how tracers reflect sources and processes by identifying different biochemical regions in an urbanised coastal environment using a generalist bioindicator, *M*. *plebejus*. This species was an effective bioindicator of δ^13^C, δ^15^N and some trace elements, such as Pb and Cu. However, discrepancies between some sediment and prawn trace element distribution patterns (e.g. Ni) indicated that local environmental conditions and prawn physiology and diet also had a measurable effect on prawn values. Therefore, juveniles were useful indicators of environmental and chemical conditions at a regional level, but the results of some tracers should be interpreted with caution. The final analysis showed it was possible to distinguish seven of the eight original sample areas within Moreton Bay. These “fingerprints” were able to help distinguish the dominant influences, i.e. urban, catchment and offshore, that affected different sample areas. The majority of tracers showed clear transitions between nearshore and offshore environments. Inshore prawn δ^15^N, Cu, and Pb values indicated prawns were exposed to higher levels of different land-based pollution. Changes in δ^13^C, Cd, and As appeared to indicate a transition from terrestrial to marine resources. However, less commonly used tracers, such as REY and Co, were required to distinguish prawns from specific embayments. Greater study of the distribution and biological uptake of less commonly used biochemical tracers (i.e. REY), could help increase our understanding of coastal regions and species. In future, continued monitoring of Moreton Bay using the strategies presented in this study would allow researchers and managers to observe and track environmental changes in relation to local conditions and development. Ultimately, tracerscapes can lead to more accurate assessments of how changes in local processes and catchment inputs may affect coastal estuaries and basins.

## Supporting information

S1 Table Analytical precision of major and trace elements measured in *M*. *plebejus* muscle tissue collected in Moreton Bay. Duplicate samples were run and precision was calculated as the mean and standard deviation of the coefficient of variance (%) for all pairs of duplicates.
(DOCX)

S2 Table Mean (standard deviation) and Z-score (standard deviation) values of *M*. *plebejus* muscle stable isotope values for each sample site in Moreton Bay. Stable isotope values are expressed as δ notation as deviations from standards in parts per thousand (‰).
(DOCX)

S3 Table Major and trace element concentrations of *M*. *plebejus* muscle composites for each sample site in Moreton Bay. Values for Al, Fe, and Mn are expressed as weight percent, all other elements are expressed as mg/kg.
(DOCX)

S4 Table Major and trace element Z-scores of *M*. *plebejus* muscle composites for each sample site in Moreton Bay. Major and trace element values from Site 23-DB were not included in Z-score calculations due to contamination concerns.
(DOCX)

## References

[1] Beck MW, Heck KL, Able KW, Childers DL, Eggleston DB, Gillanders BM, et al The identification, conservation, and management of estuarine and marine nurseries for fish and invertebrates. BioScience. 2001;51:633–41. 10.1641/0006-3568(2001)051[0633:ticamo]2.0.co;2

[2] Nixon SW, Oviatt CA, Frithsen J, Sullivan B. Nutrients and the productivity of estuarine and coastal marine ecosystems. J Limnol Soc South Africa. 1986;12:43–71. 10.1080/03779688.1986.9639398

[3] Spalding MD, Fox HE, Allen GR, Davidson N, Ferdaña ZA, Finlayson M, et al Marine ecoregions of the world: a bioregionalization of coastal and shelf areas. BioScience. 2007;57:573–83. 10.1641/b570707

[4] Barbier EB, Hacker SD, Kennedy C, Koch EW, Stier AC, Silliman BR. The value of estuarine and coastal ecosystem services. Ecol Monograph. 2011;81:169–93. 10.1890/10-1510.1

[5] Wang Y, Ridd PV, Heron ML, Stieglitz TC, Orpin AR. Flushing time of solutes and pollutants in the central Great Barrier Reef lagoon, Australia. Mar Fresh Res. 2007;58:778–91. 10.1071/MF06148

[6] Devlin MJ, Brodie J. Terrestrial discharge into the Great Barrier Reef Lagoon: nutrient behavior in coastal waters. Mar Poll Bull. 2005;51:9–22. 10.1016/j.marpolbul.2004.10.037 15757704

[7] Lapointe BE, Clark MW. Nutrient inputs from the watershed and coastal eutrophication in the Florida Keys. Estuaries. 1992;15:465–76. 10.2307/1352391

[8] Mallin MA, Paerl HW, Rudek J, Bates PW. Regulation of estuarine primary production by watershed rainfall and river flow. Mar Ecol Prog Ser. 1993:199–203.

[9] Lotze HK, Lenihan HS, Bourque BJ, Bradbury RH, Cooke RG, Kay MC, et al Depletion, degradation, and recovery potential of estuaries and coastal seas. Science. 2006;312:1806–9. 10.1126/science.1128035 16794081

[10] Forrest BM, Gillespie PA, Cornelisen CD, Rogers KM. Multiple indicators reveal river plume influence on sediments and benthos in a New Zealand coastal embayment. New Zeal J Mar Fresh. 2007;41:13–24. 10.1080/00288330709509892

[11] Connolly RM, Gorman D, Guest MA. Movement of carbon among estuarine habitats and its assimilation by invertebrates. Oecologia. 2005;144:684–91. 10.1007/s00442-005-0167-4 16001216

[12] Viana IG, Bode A. Stable nitrogen isotopes in coastal macroalgae: Geographic and anthropogenic variability. Sci Total Environ. 2013;443:887–95. 10.1016/j.scitotenv.2012.11.065 23247291

[13] Prego R, Caetano M, Bernárdez P, Brito P, Ospina-Alvarez N, Vale C. Rare earth elements in coastal sediments of the northern Galician shelf: Influence of geological features. Cont Shelf Res. 2012;35:75–85. 10.1016/j.csr.2011.12.010

[14] Walther BD, Nims MK. Spatiotemporal variation of trace elements and stable isotopes in subtropical estuaries: I. freshwater endmembers and mixing curves. Estuar Coast. 2015;38:754–68. 10.1007/s12237-014-9881-7

[15] Phillips DL, Gregg JW. Source partitioning using stable isotopes: coping with too many sources. Oecologia. 2003;136:261–9. 10.1007/s00442-003-1218-3 12759813

[16] Fry B. Alternative approaches for solving underdetermined isotope mixing problems. Mar Ecol Prog Ser. 2013;472:1–13. 10.3354/meps10168

[17] Bouillon S, Connolly R, Gillikin D. Use of stable isotopes to understand food webs and ecosystem functioning in estuaries In: Wolanski E, McLusky D, editors. Treatise on Estuarine and Coastal Science. 7 Waltham: Academic Press; 2011 p. 143–73.

[18] Connolly RM, Waltham NJ. Spatial analysis of carbon isotopes reveals seagrass contribution to fishery food web. Ecosphere. 2015;6:1–12. 10.1890/ES14-00243.1

[19] Brett MT. Resource polygon geometry predicts Bayesian stable isotope mixing model bias. Mar Ecol Prog Ser. 2014;514:1–12. 10.3354/meps11017

[20] Carson HS, Morgan SG, Green PG. Fine-scale chemical fingerprinting of an open coast crustacean for the assessment of population connectivity. Mar Biol. 2008;153:327–35. 10.1007/s00227-007-0808-8

[21] Zhou Q, Zhang J, Fu J, Shi J, Jiang G. Biomonitoring: An appealing tool for assessment of metal pollution in the aquatic ecosystem. Anal Chim Acta. 2008;606:135–50. 10.1016/j.aca.2007.11.018 18082645

[22] Markert B, Wappelhorst O, Weckert V, Herpin U, Siewers U, Friese K, et al The use of bioindicators for monitoring the heavy-metal status of the environment. J Radioanal Nucl Chem. 1999;240:425–9. 10.1007/bf02349387

[23] Rainbow PS. Ecophysiology of trace metal uptake in crustaceans. Estuar Coast Shelf Sci. 1997;44:169–76. 10.1006/ecss.1996.0208

[24] Lebrun JD, Perret M, Uher E, Tusseau-Vuillemin M-H, Gourlay-Francé C. Waterborne nickel bioaccumulation in *Gammarus pulex*: Comparison of mechanistic models and influence of water cationic composition. Aquat Toxicol. 2011;104:161–7. 10.1016/j.aquatox.2011.04.011 21632021

[25] Stankovic S, Kalaba P, Stankovic AR. Biota as toxic metal indicators. 2014;12:63–84. 10.1007/s10311-013-0430-6

[26] Soto-Jiménez MF, Páez-Osuna F, Scelfo G, Hibdon S, Franks R, Aggarawl J, et al Lead pollution in subtropical ecosystems on the SE Gulf of California Coast: A study of concentrations and isotopic composition. Mar Environ Res. 2008;66:451–8. 10.1016/j.marenvres.2008.07.009 18789522

[27] Lewtas KLM, Birch GF, Foster-Thorpe C. Metal accumulation in the greentail prawn, *Metapenaeus bennettae*, in Sydney and Port Hacking estuaries, Australia. Environ Sci Pollut Res. 2014;21:704–16. 10.1007/s11356-013-1961-x 23852467

[28] Fry B, Baltz D, Benfield M, Fleeger J, Gace A, Haas H, et al Stable isotope indicators of movement and residency for brown shrimp (*Farfantepenaeus aztecus*) in coastal Louisiana marshscapes. Estuaries. 2003;26:82–97. 10.1007/bf02691696

[29] Fry B, Arnold C. Rapid ^13^C/^12^C turnover during growth of brown shrimp (*Penaeus aztecus*). Oecologia. 1982;54:200–4. 10.1007/BF00378393 28311429

[30] Gamboa-Delgado J, Peña-Rodríguez A, Ricque-Marie D, Cruz-Suárez LE. Assessment of Nutrient Allocation and Metabolic Turnover Rate in Pacific White Shrimp Litopenaeus vannamei Co-Fed Live Macroalgae Ulva clathrata and Inert Feed: Dual Stable Isotope Analysis. 2011;30:969–78. 10.2983/035.030.0340

[31] Suthers I. Functional morphology of the mouthparts and gastric mill in *Penaeus plebejus* Hess (Decapoda: Penaeidea). Mar Fresh Res. 1984;35:785–92.

[32] Racek AA. Prawn investigations in eastern Australia. N.S.W., Australia: Res. Bull. State. Fish.; 1959.

[33] Moriarty D. Quantification of carbon, nitrogen and bacterial biomass in the food of some penaeid prawns. Mar Fresh Res. 1977;28:113–8.

[34] Courtney A, Kienzle M, Pascoe S, O'Neill M, Leigh GM, Wang Y-G, et al Harvest strategy evaluations and co-management for the Moreton Bay Trawl Fishery: Australian Seafood Cooperative Research Centre Bedford Park; 2012.

[35] Courtney A, Masel J, Die D. Temporal and spatial patterns in recruitment of three penaeid prawns in Moreton Bay, Queensland, Australia. Estuar Coast Mar Sci. 1995;41:377–92.

[36] Eyre B, Hossain S, McKee L. A suspended sediment budget for the modified subtropical Brisbane River estuary, Australia. Estuar Coast Mar Sci. 1998;47:513–22. 10.1006/ecss.1998.0371

[37] Coates-Marnane J, Olley J, Burton J, Grinham A. The impact of a high magnitude flood on metal pollution in a shallow subtropical estuarine embayment. Sci Total Environ. 2016;569:716–31. 10.1016/j.scitotenv.2016.06.193 27380395

[38] Water HLa. Ecosystem Health Monitoring Program 2018.

[39] Healthy Waterways Report Card 2017: Healthy Waterways; 2017 [cited 2017 2 November]. Available from: http://healthywaterways.org/reportcard#/overview/condition

[40] Ruello N, editor An historical review and annotated bibliography of prawns and the prawning industry in Australia First Australian National Prawn Seminar; 1975; Canberra, Australia: Australian Government Printing Service.

[41] Kirkegaard I, Walker RH. Synopsis of biological data on the Eastern King Prawn *Penaeus plebejus* Hess, 1865. Melbourne, Australia: Division of Fisheries and Oceanography CSIRO Australia; 1970.

[42] Glaister J, Montgomery S, McDonall V. Yield-per-recruit analysis of eastern king prawns *Penaeus plebejus* Hess, in eastern Australia. Mar Fresh Res. 1990;41:175–97. 10.1071/MF9900175

[43] Montgomery S. Tagging studies on juvenile eastern king prawns reveal record migration. Aust Fish. 1981;40:13–4.

[44] Montgomery SS. Movements of juvenile eastern king prawns, *Penaeus plebejus*, and identification of stock along the east coast of Australia. Fish Res 1990;9:189–208. 10.1016/S0165-7836(05)80001-3

[45] Ruello N. Geographical distribution, growth and breeding miagration of the eastern Australian king prawn *Penaeus plebejus* Hess. Mar Fresh Res. 1975;26:343–54. 10.1071/MF9750343.

[46] Rothlisberg P, Craig P, Andrewartha J. Modelling penaeid prawn larval advection in Albatross Bay, Australia: defining the effective spawning population. Mar Fresh Res. 1996;47:157–68. 10.1071/MF9960157

[47] Rothlisberg PC, Church JA. Processes controlling the larval dispersal and postlarval recruitment of penaeid prawns In: Sammarco PW, Heron ML, editors. The Bio-Physics of Marine Larval Dispersal. Coastal and Estuarine Studies: American Geophysical Union; 2013 p. 235–52.

[48] Young P. Moreton Bay, Queensland: a nursery area for juvenile penaeid prawns. Mar Fresh Res. 1978;29:55–75. 10.1071/MF9780055

[49] Masel J, Smallwood D. Habitat usage by postlarval and juvenile prawns in Moreton Bay, Queensland, Australia. Proc R Soc Queensl. 2000;109:107.

[50] Glaister J, Lau T, McDonall V. Growth and migration of tagged eastern Australian king prawns, *Penaeus plebejus* Hess. Mar Fresh Res. 1987;38:225–41. 10.1071/MF9870225.

[51] Fry B, Carter JF, Tinggi U, Arman A, Kamal M, Metian M, et al Prawn biomonitors of nutrient and trace metal pollution along Asia-Pacific coastlines. Isotopes Environ Health Stud. 2016;52:619–32. 10.1080/10256016.2016.1149481 26982881

[52] Pourang N, Dennis J, Ghourchian H. Tissue distribution and redistribution of trace elements in shrimp species with the emphasis on the roles of metallothionein. Ecotoxicology. 2004;13:519–33. 15526858 10.1023/b:ectx.0000037189.80775.9c

[53] Wang W-X, Fisher NS. Delineating metal accumulation pathways for marine invertebrates. Sci Total Environ. 1999;237:459–72.

[54] Rainbow PS. Trace metal bioaccumulation: Models, metabolic availability and toxicity. Environ Int. 2007;33:576–82. 10.1016/j.envint.2006.05.007. 16814385

[55] Cresswell T, Mazumder D, Callaghan PD, Nguyen A, Corry M, Simpson SL. Metal transfer among organs following short-and long-term exposures using autoradiography: cadmium bioaccumulation by the freshwater prawn *Macrobrachium australiense*. Environ Sci Technol. 2017;51:4054–60. 10.1021/acs.est.6b06471 28299929

[56] Jackson AL, Inger R, Parnell AC, Bearhop S. Comparing isotopic niche widths among and within communities: SIBER—Stable Isotope Bayesian Ellipses in R. J Anim Ecol. 2011;80:595–602. 10.1111/j.1365-2656.2011.01806.x WOS:000289160900011. 21401589

[57] Parnell A, Jackson A. siar: Stable Isotope Analysis in R. R package version 4.2. http://CRANR-projectorg/package=siar2013.

[58] Weihs C, Ligges U, Luebke K, Raabe N. klaR analyzing german business cycles In: Baier D, Decker R, Schmidt-Thieme L, editors. Data analysis and decision support. Berlin: Springer-Verlag 2005 p. 335–43.

[59] Savage C. Tracing the influence of sewage nitrogen in a coastal ecosystem using stable nitrogen isotopes. Ambio. 2005;34:145–50. 15865312

[60] Costanzo SD, O’Donohue MJ, Dennison WC, Loneragan NR, Thomas M. A new approach for detecting and mapping sewage impacts. Mar Poll Bull. 2001;42:149–56. 10.1016/S0025-326X(00)00125-9 11381886

[61] Costanzo SD, Udy J, Longstaff B, Jones A. Using nitrogen stable isotope ratios (δ15N) of macroalgae to determine the effectiveness of sewage upgrades: changes in the extent of sewage plumes over four years in Moreton Bay, Australia. Mar Poll Bull. 2005;51:212–7. 10.1016/j.marpolbul.2004.10.018 15757722

[62] Páez-Osuna F, Ruiz-Fernández C. Trace metals in the Mexican shrimp Penaeus vannamei from estuarine and marine environments. Environ Pollut. 1995;87:243–7. 10.1016/0269-7491(94)P2612-D. 15091599

[63] Páez-Osuna F, Ruiz-Fernandez C. Comparative bioaccumulation of trace metals in *Penaeus stylirostris* in estuarine and coastal environments. Estuar Coast Shelf Sci. 1995;40:35–44.

[64] Nascimento JR, Sabadini-Santos E, Carvalho C, Keunecke KA, César R, Bidone ED. Bioaccumulation of heavy metals by shrimp (*Litopenaeus schmitti*): A dose–response approach for coastal resources management. Mar Poll Bull. 2017;114:1007–13. 10.1016/j.marpolbul.2016.11.013. 27876373

[65] Wassenberg T, Hill B. Natural diet of the tiger prawns *Penaeus esculentus* and *P*. *semisulcatus*. Mar Fresh Res. 1987;38:169–82. 10.1071/MF9870169.

[66] Stephenson RL, Lyon GL. Carbon-13 depletion in an estuarine bivalve: detection of marine and terrestrial food sources. Oecologia. 1982;55:110–3. 10.1007/BF00386725 28309909

[67] Connolly RM, Schlacher TA, Gaston† TF. Stable isotope evidence for trophic subsidy of coastal benthic fisheries by river discharge plumes off small estuaries. Mar Biol Res. 2009;5:164–71. 10.1080/17451000802266625

[68] Roelfsema C, Kovacs EM, Saunders MI, Phinn S, Lyons M, Maxwell P. Challenges of remote sensing for quantifying changes in large complex seagrass environments. Estuar Coast Shelf Sci. 2013;133:161–71. 10.1016/j.ecss.2013.08.026

[69] Pitt KA, Connolly RM, Maxwell P. Redistribution of sewage-nitrogen in estuarine food webs following sewage treatment upgrades. Mar Poll Bull. 2009;58:573–80. 10.1016/j.marpolbul.2008.11.016 19138774

[70] Eyre BD, McKee LJ. Carbon, nitrogen, and phosphorus budgets for a shallow subtropical coastal embayment (Moreton Bay, Australia). Limnol Oceanogr. 2002;47:1043–55. 10.4319/lo.2002.47.4.1043

[71] Savage C, Leavitt PR, Elmgren R. Distribution and retention of effluent nitrogen in surface sediments of a coastal bay. Limnol Oceanogr. 2004;49:1503–11. 10.4319/lo.2004.49.5.1503

[72] Altabet MA. Isotopic tracers of the marine nitrogen cycle: present and past In: Volkman JK, editor. Marine organic matter: biomarkers, isotopes and DNA. 2N. Berlin, Heidelberg: Springer; 2006 p. 251–93.

[73] Brandes JA, Devol AH. Isotopic fractionation of oxygen and nitrogen in coastal marine sediments. Geochim Cosmochim Acta 1997;61:1793–801. 10.1016/S0016-7037(97)00041-0

[74] Burford MA, Green SA, Cook AJ, Johnson SA, Kerr JG, O’Brien KR. Sources and fate of nutrients in a subtropical reservoir. Aquat Sci. 2012;74:179–90. 10.1007/s00027-011-0209-4

[75] Connolly RM, Guest MA, Melville AJ, Oakes JM. Sulfur stable isotopes separate producers in marine food-web analysis. Oecologia. 2004;138:161–7. 10.1007/s00442-003-1415-0 14593525

[76] Newell RIE, Marshall N, Sasekumar A, Chong VC. Relative importance of benthic microalgae, phytoplankton, and mangroves as sources of nutrition for penaeid prawns and other coastal invertebrates from Malaysia. Mar Biol. 1995;123:595–606. 10.1007/bf00349238

[77] Fry B, Scalan RS, Winters JK, Parker PL. Sulphur uptake by salt grasses, mangroves, and seagrasses in anaerobic sediments. Geochim Cosmochim Acta. 1982;46:1121–4. 10.1016/0016-7037(82)90063-1

[78] Peterson BJ. Stable isotopes as tracers of organic matter input and transfer in benthic food webs: A review. Acta Oecol. 1999;20:479–87. 10.1016/S1146-609X(99)00120-4

[79] Oakes JM, Connolly RM. Causes of sulfur isotope variability in the seagrass, *Zostera capricorni*. J Exp Mar Bio Ecol. 2004;302:153–64. 10.1016/j.jembe.2003.10.011

[80] Bolnick DI, Yang LH, Fordyce JA, Davis JM, Svanback R. Measuring individual-level resource specialisation. Ecology. 2002:2936–41. 10.1890/0012-9658(2002)083[2936:MILRS]2.0.CO;2

[81] Bolnick DI, Svanbäck R, Araújo MS, Persson L. Comparative support for the niche variation hypothesis that more generalized populations also are more heterogeneous. Proc Nat Acad Sci. 2007;104:10075–9. 10.1073/pnas.0703743104 17537912 PMC1891261

[82] Cox ME, Preda M. Trace metal distribution within marine and estuarine sediments of western Moreton Bay, Queensland, Australia: relation to land use and setting. Geog Res. 2005;43:173–93. 10.1111/j.1745-5871.2005.00312.x

[83] Tchounwou PB, Yedjou CG, Patlolla AK, Sutton DJ. Heavy metals toxicity and the environment In: Luch A, editor. Molecular, Clinical and Environmental Toxicology. Experientia Supplementum. 101 Basel: Springer; 2012 p. 133–64. 10.1007/978-3-7643-8340-4_6 PMC4144270 22945569

[84] Prosi F. Factors controlling biological availability and toxic effects of lead in aquatic organisms. Sci Total Environ. 1989;79:157–69. 10.1016/0048-9697(89)90359-8 2727669

[85] Arzul G, Maguer J-F. Influence of pig farming on the copper content of estuarine sediments in Brittany, France. Mar Poll Bull. 1990;21:431–4. 10.1016/0025-326X(90)90762-W

[86] Hargreaves JC, Adl MS, Warman PR. A review of the use of composted municipal solid waste in agriculture. Agric Ecosyst Environ. 2008;123:1–14. 10.1016/j.agee.2007.07.004

[87] Monbet P Tagging studies on juvenile eastern king prawns reveal record migration. Dissolved and particulate fluxes of copper through the Morlaix river estuary (Brittany, France): mass balance in a small estuary with strong agricultural catchment. Mar Poll Bull. 2004;48:78–86. 10.1016/S0025-326X(03)00327-8 14725877

[88] Claisse D, Alzieu C. Copper contamination as a result of antifouling paint regulations? Mar Poll Bull. 1993;26:395–7. 10.1016/0025-326X(93)90188-P

[89] Schiff K, Diehl D, Valkirs A. Copper emissions from antifouling paint on recreational vessels. Mar Poll Bull. 2004;48:371–7. 10.1016/j.marpolbul.2003.08.016 14972590

[90] Turner A. Marine pollution from antifouling paint particles. Mar Poll Bull. 2010;60:159–71. 10.1016/j.marpolbul.2009.12.004 20060546

[91] Barwick M, Maher W. Biotransference and biomagnification of selenium copper, cadmium, zinc, arsenic and lead in a temperate seagrass ecosystem from Lake Macquarie Estuary, NSW, Australia. Mar Environ Res. 2003;56:471–502. 10.1016/S0141-1136(03)00028-X 12860434

[92] Carter JF, Tinggi U, Yang X, Fry B. Stable isotope and trace metal compositions of Australian prawns as a guide to authenticity and wholesomeness. Food Chem. 2015;170:241–8. 10.1016/j.foodchem.2014.08.037 25306341

[93] Ruelas-Inzunza J, Green-Ruiz C, Zavala-Nevárez M, Soto-Jiménez M. Biomonitoring of Cd, Cr, Hg and Pb in the Baluarte River basin associated to a mining area (NW Mexico). Sci Total Environ. 2011;409:3527–36. 10.1016/j.scitotenv.2011.05.035 21684575

[94] Roy P, Crawford E. Heavy metals in a contaminated Australian estuary—dispersion and accumulation trend. Estuar Coast Shelf Sci. 1984;19:341–58. 10.1016/0272-7714(84)90030-1

[95] Sanders JG, Windom HL. The uptake and reduction of arsenic species by marine algae. Estuar Coast Mar Sci. 1980;10:555–67. 10.1016/S0302-3524(80)80075-2

[96] Riedel GF. The annual cycle of arsenic in a temperate estuary. Estuaries. 1993;16:533–40. 10.2307/1352600

[97] Neff JM. Ecotoxicology of arsenic in the marine environment. Environ Toxicol Chem. 1997;16:917–27. 10.1002/etc.5620160511

[98] Zhang W, Wang W-X, Zhang L. Arsenic speciation and spatial and interspecies differences of metal concentrations in mollusks and crustaceans from a South China estuary. Ecotoxicology. 2013;22:671–82. 10.1007/s10646-013-1059-8 23475307

[99] Price A, Maher W, Kirby J, Krikowa F, Duncan E, Taylor A, et al Distribution of arsenic species in an open seagrass ecosystem: relationship to trophic groups, habitats and feeding zones. Environ Chem. 2012;9:77–88. 10.1071/EN11105

[100] Tukai R, Maher WA, McNaught IJ, Ellwood MJ, Coleman M. Occurrence and chemical form of arsenic in marine macroalgae from the east coast of Australia. Mar Fresh Res. 2002;53:971–80. 10.1071/MF01230

[101] Byrd JT. The seasonal cycle of arsenic in estuarine and nearshore waters of the South Atlantic Bight. Mar Chem. 1988;25:383–94. 10.1016/0304-4203(88)90118-1

[102] Dobson S, Stewart I, Kiermeier A, Rogers S, McLeod C. Risk assessment of cadmium in Australian wild-caught prawn muscle tissue Australian Seafood Cooperative Research Centre 2014 Contract No.: 2009/787.

[103] Rainbow PS. Physiology, physicochemistry and metal uptake—A crustacean perspective. Mar Poll Bull. 1995;31:55–9. 10.1016/0025-326X(95)00005-8

[104] Rainbow PS, Amiard-Triquet C, Amiard JC, Smith BD, Langston WJ. Observations on the interaction of zinc and cadmium uptake rates in crustaceans (amphipods and crabs) from coastal sites in UK and France differentially enriched with trace metals. Aquat Toxicol. 2000;50:189–204. 10.1016/S0166-445X(99)00103-4 10958954

[105] Buchwalter DB, Luoma SN. Differences in dissolved cadmium and zinc uptake among stream insects: mechanistic explanations. Environ Sci Technol. 2005;39:498–504. 10.1021/es0404421 15707049

[106] Nugegoda D, Rainbow PS. The uptake of dissolved zinc and cadmium by the decapod crustacean *Palaemon elegans*. Mar Poll Bull. 1995;31:460–3. 10.1016/0025-326X(95)00131-6

[107] Munksgaard NC, Burchert S, Kaestli M, Nowland SJ, O'Connor W, Gibb KS. Cadmium uptake and zinc-cadmium antagonism in Australian tropical rock oysters: Potential solutions for oyster aquaculture enterprises. Mar Poll Bull. 2017;123:47–56. 10.1016/j.marpolbul.2017.09.031 28938999

[108] Lawrence MG, Kamber BS. The behaviour of the rare earth elements during estuarine mixing—revisited. Mar Chem. 2006;100:147–61. 10.1016/j.marchem.2005.11.007

[109] Elderfield H, Upstill-Goddard R, Sholkovitz E. The rare earth elements in rivers, estuaries, and coastal seas and their significance to the composition of ocean waters. Geochim Cosmochim Acta. 1990;54:971–91. 10.1016/0016-7037(90)90432-K

[110] Herrmann H, Nolde J, Berger S, Heise S. Aquatic ecotoxicity of lanthanum–A review and an attempt to derive water and sediment quality criteria. Ecotoxicol Environ Saf. 2016;124:213–38. 10.1016/j.ecoenv.2015.09.033 26528910

[111] Ramesh R, Ramanathan A, James RA, Subramanian V, Jacobsen S, Holland H. Rare earth elements and heavy metal distribution in estuarine sediments of east coast of India. Hydrobiol. 1999;397:89–99. 10.1023/A:1003646631589

[112] Lawrence MG, Greig A, Collerson KD, Kamber BS. Rare earth element and yttrium variability in south east Queensland waterways. Aquat Geochem. 2006;12:39–72. 10.1007/s10498-005-4471-8

[113] Moermond CT, Tijink J, Van Wezel AP, Koelmans AA. Distribution, speciation, and bioavailability of lanthanides in the Rhine‐Meuse estuary, The Netherlands. Environ Toxicol Chem. 2001;20:1916–26. 10.1002/etc.5620200909 11521817

[114] Shynu R, Rao VP, Kessarkar PM, Rao T. Rare earth elements in suspended and bottom sediments of the Mandovi estuary, central west coast of India: Influence of mining. Estuar Coast Shelf Sci. 2011;94:355–68. 10.1016/j.ecss.2011.07.013

[115] Hannigan R, Dorval E, Jones C. The rare earth element chemistry of estuarine surface sediments in the Chesapeake Bay. Chem Geol. 2010;272:20–30.

[116] Munroe S, Fry B, Olley J. Underutilized biogeochemical tracers distinguish invertebrate populations in a complex river system. 2018;16:444–58. 10.1002/lom3.10258

[117] Xu S, Lin C, Qiu P, Song Y, Yang W, Xu G, et al Tungsten- and cobalt-dominated heavy metal contamination of mangrove sediments in Shenzhen, China. Mar Poll Bull. 2015;100:562–6. 10.1016/j.marpolbul.2015.08.031 26323860

[118] Barrio-Parra F, Elío J, De Miguel E, García-González JE, Izquierdo M, Álvarez R. Environmental risk assessment of cobalt and manganese from industrial sources in an estuarine system. Environ Geochem Health. 2017 10.1007/s10653-017-0020-9 28861663

[119] Núñez-Nogueira G, Fernández-Bringas L, Ordiano-Flores A, Gómez-Ponce A. Ni accumulation and regulation after experimental exposure to a Cd, Pb, and Zn mixture in the Pacific white shrimp *Penaeus vannamei*. Water Air Soil Pollut. 2013;224:1644 10.1007/s11270-013-1644-8

[120] Blewett TA, Glover CN, Fehsenfeld S, Lawrence MJ, Niyogi S, Goss GG, et al Making sense of nickel accumulation and sub-lethal toxic effects in saline waters: Fate and effects of nickel in the green crab, *Carcinus maenas*. Aquat Toxicol. 2015;164:23–33. 10.1016/j.aquatox.2015.04.010 25914092

[121] Blewett TA, Leonard EM. Mechanisms of nickel toxicity to fish and invertebrates in marine and estuarine waters. Environ Pollut. 2017;223:311–22. 10.1016/j.envpol.2017.01.028 28122673

[122] Ünsal M. The accumulation and transfer of vanadium within the food chain. Mar Poll Bull. 1982;13:139–41. 10.1016/0025-326X(82)90373-3

[123] Kustin K, McLeod GC, Gilbert TR, Le Briggs BR. Vanadium and other metal ions in the physiological ecology of marine organisms In: Clarke MJ, editor. Copper, molybdenum, and vanadium in biological systems. Structure and Bonding. Berlin, Heidelberg: Springer-Verlag; 1983 p. 139–60.

[124] Odate S, Pawlik JR. The role of vanadium in the chemical defense of the solitary tunicate, *Phallusia nigra*. J Chem Ecol. 2007;33:643–54. 10.1007/s10886-007-9251-z 17265174

[125] Ünsal M. Transfer pathways and accumulation of vanadium in the crab *Carcinus maenas*. Mar Biol. 1983;72:279–82. 10.1007/bf00396833

[126] Liu Y, Zhou Q, Xu J, Xue Y, Liu X, Wang J, et al Assessment of total and organic vanadium levels and their bioaccumulation in edible sea cucumbers: tissues distribution, inter-species-specific, locational differences and seasonal variations. Environ Geochem Health. 2016;38:111–22. 10.1007/s10653-015-9689-9 25732906

[127] Miramand P, Guary JC, Fowler SW. Uptake, assimilation, and excretion of vanadium in the shrimp, *Lysmata seticaudata* (Risso), and the crab, *Carcinus maenas* (L.). J Exp Mar Bio Ecol. 1981;49:267–87. 10.1016/0022-0981(81)90076-9

[128] Hamilton EI. The geobiochemistry of cobalt. Sci Total Environ. 1994;150:7–39. 10.1016/0048-9697(94)90126-0 7939612

[129] Rainbow PS. Trace metal concentrations in aquatic invertebrates: why and so what? Environ Pollut. 2002;120:497–507. 10.1016/S0269-7491(02)00238-5 12442773

