# Supplementary material for: A benthic bioindicator reveals distinct land and ocean–Based influences in an urbanized coastal embayment
Source: PLoS One. 2018 Oct 11;13(10):e0205408. doi: 10.1371/journal.pone.0205408 (PMC6181360; doi:10.1371/journal.pone.0205408)
Supplement: S1 Table — Duplicate samples were run and precision was calculated as the mean and standard deviation of the coefficient of variance (%) for all pairs of duplicates. (DOCX) [file pone.0205408.s001.docx]

**S1 Table.** **Analytical precision of major and trace elements measured in M. plebejus muscle tissue collected in Moreton Bay.** Duplicate samples were run and precision was calculated as the mean and standard deviation of the coefficient of variance (%) for all pairs of duplicates.

| **Trace Element** | **Mean (±SD)** |
| --- | --- |
| **Al** | 12.6 (7.3) |
| **As** | 7.1 (6.4) |
| **Cd** | 6.6 (4.06) |
| **Ce** | 7.2 (9.8) |
| **Co** | 11.9 (10.5) |
| **Cu** | 6.1 (5.4) |
| **Fe** | 4.8 (5.8) |
| **La** | 4.5 (6.7) |
| **Mn** | 5.5 (6.3) |
| **Ni** | 22.1 (7.5) |
| **Pb** | 6.6 (5.7) |
| **V** | 8.5 (14.4) |
| **Y** | 11.6 (13.7) |
