# Supplementary material for: A benthic bioindicator reveals distinct land and ocean–Based influences in an urbanized coastal embayment
Source: PLoS One. 2018 Oct 11;13(10):e0205408. doi: 10.1371/journal.pone.0205408 (PMC6181360; doi:10.1371/journal.pone.0205408)
Supplement: S2 Table — Stable isotope values are expressed as δ notation as deviations from standards in parts per thousand (‰). (DOCX) [file pone.0205408.s002.docx]

**S2 Table. Mean (standard deviation) and Z-score (standard deviation) values of M. plebejus muscle stable isotope values for each sample site in Moreton Bay.** Stable isotope values are expressed as δ notation as deviations from standards in parts per thousand (‰).

| Site | Isotopes |  |  | Z-scores |  |  |
| --- | --- | --- | --- | --- | --- | --- |
|  | **δ^15^N** | **δ^13^C** | **δ^34^S** | **δ^15^N** | **δ^13^C** | **δ^34^S** |
| 1-Off | 7.98 (0.91) | -14.90 (1.01) | 15.76 (1.17) | -0.75 (0.38) | 0.38 (0.51) | 0.34 (0.58) |
| 2-Off | 7.04 (0.86) | -14.22 (1.42) | 13.79 (2.92) | -1.14 (0.36) | 0.74 (0.72) | -0.64 (1.46) |
| 3-Off | 7.32 (0.88) | -13.69 (1.14) | 14.81 (2.39) | -1.19 (0.37) | 1.00 (0.58) | -0.12 (1.20) |
| 4-Off | 7.46 (0.93) | -12.85 (1.95) | 14.41 (2.90) | -0.97 (0.39) | 1.42 (0.99) | -0.32 (1.46) |
| 5-Off | 7.94 (1.51) | -15.63 (2.25) | 15.22 (2.74) | -0.76 (0.64) | 0.02 (1.14) | 0.08 (1.38) |
| 6-DB | 10.64 (2.89) | -16.29 (2.24) | 14.66 (1.55) | 0.36 (1.23) | -0.32 (1.13) | -0.15 (0.77) |
| 7-DB | 11.20 (1.65) | -16.76 (1.03) | 16.17 (0.66) | 0.61 (0.70) | -0.55 (0.52) | 0.55 (0.33) |
| 8-DB | 9.13 (1.78) | -16.69 (1.91) | 14.97 (1.65) | -0.29 (0.76) | -0.51 (0.97) | -0.11 (0.83) |
| 23-DB | 11.48 (0.71) | -18.19 (0.79) | 16.71 (0.58) | 0.73 (0.30) | -1.28 (0.40) | 0.82 (0.29) |
| 9-BB | 13.53 (0.90) | -18.09 (0.51) | 16.63 (0.68) | 1.60 (0.38) | -1.23 (0.25) | 0.78 (0.34) |
| 10-BB | 12.62 (1.63) | -16.50 (1.45) | 15.76 (0.36) | 1.22 (0.69) | -0.42 (0.73) | 0.27 (0.18) |
| 11-BB | 13.39 (1.93) | -17.26 (1.19) | 14.55 (1.33) | 1.50 (0.82) | -0.79 (0.60) | -0.26 (0.67) |
| 12-CB | 10.12 (2.97) | -15.32 (2.82) | 13.93 (3.2) | 0.16 (1.26) | 0.17 (1.43) | -0.56 (1.63) |
| 13-CB | 9.87 (2.23) | -15.81 (1.95) | 15.13 (2.59) | 0.04 (0.94) | -0.04 (0.99) | 0.06 (1.3) |
| 14-EB | 8.10 (1.91) | -13.82 (2.27) | 14.00 (2.77) | -0.70 (0.81) | 0.93 (0.15) | -0.54 (1.39) |
| 15-EB | 9.26 (1.64) | -15.51 (1.66) | 15.31 (1.22) | -0.24 (0.70) | 0.07 (0.84) | 0.10 (0.61) |
| 16-CS | 10.86 (1.34) | -16.27 (0.98) | 15.06 (0.78) | 0.46 (0.57) | -0.28 (0.49) | 0.05 (0.39) |
| 17-CS | 9.21 (1.27) | -15.33 (1.59) | 15.90 (1.49) | -0.22 (0.54) | 0.17 (0.81) | 0.41 (0.75) |
| 18-CS | 9.74 (2.16) | -15.71 (2.05) | 15.39 (2.5) | 0.001 (0.92) | -0.02 (1.04) | 0.15 (1.28) |
| 19-WB | 10.67 (1.02) | -15.70 (0.76) | 14.88 (1.33) | 0.37 (0.43) | -0.03 (0.38) | -0.09 (0.67) |
| 20-SB | 7.91 (0.60) | -15.49 (0.96) | 13.29 (0.94) | -0.81 (0.25) | 0.08 (0.49) | -0.87 (0.47) |
| 21-SB | 9.57 (0.99) | -14.59 (1.05) | 14.29 (1.30) | -0.11 (0.42) | 0.54 (0.82) | -0.31 (0.65) |
| 22-SB | 9.05 (0.81) | -15.51 (0.79) | 15.70 (1.02) | -0.29 (0.34) | 0.07 (0.53) | 0.32 (0.29) |
