# Supplementary material for: A benthic bioindicator reveals distinct land and ocean–Based influences in an urbanized coastal embayment
Source: PLoS One. 2018 Oct 11;13(10):e0205408. doi: 10.1371/journal.pone.0205408 (PMC6181360; doi:10.1371/journal.pone.0205408)
Supplement: S3 Table — Values for Al, Fe, and Mn are expressed as weight percent, all other elements are expressed as mg/kg. (DOCX) [file pone.0205408.s003.docx]

**S3 Table. Major and trace element concentrations of M. plebejus muscle composites for each sample site in Moreton Bay.** Values for Al, Fe, and Mn are expressed as weight percent, all other elements are expressed as mg/kg.

| **Site** | **Al** | **As** | **Cd** | **Ce** | **Co** | **Cu** | **Fe** | **La** | **Mn** | **Ni** | **Pb** | **V** | **Y** |
| --- | --- | --- | --- | --- | --- | --- | --- | --- | --- | --- | --- | --- | --- |
| 1-Off | 5.1 | 16.8 | 0.44 | 0.0072 | 0.115 | 0.5 | 6.64 | 0.0082 | 0.73 | 0.175 | 0.05 | 0.10 | 0.005 |
| 2-Off | 17.5 | 12.6 | 0.65 | 0.0239 | 0.032 | 9.72 | 13.2 | 0.0114 | 1.03 | 0.095 | 0.02 | 0.07 | 0.0037 |
| 3-Off | 23.6 | 14.4 | 0.60 | 0.019 | 0.057 | 17 | 15.3 | 0.01 | 1 | 0.42 | 0.03 | 0.19 | 0.0052 |
| 4-Off | 45.5 | 15.5 | 0.94 | 0.055 | 0.053 | 13 | 26.7 | 0.026 | 1.6 | 0.45 | 0.05 | 0.26 | 0.01 |
| 5-Off | 15.2 | 11.0 | 1.87 | 0.0147 | 0.057 | 9.02 | 14.95 | 0.0098 | 1.19 | 0.19 | 0.04 | 0.16 | 0.0045 |
| 6-DB | 87.4 | 4.1 | 0.19 | 0.171 | 0.084 | 39.8 | 68.5 | 0.0975 | 3.67 | 0.20 | 0.10 | 0.16 | 0.0416 |
| 7-DB | 73.4 | 8.9 | 0.13 | 0.06 | 0.108 | 42.0 | 53.8 | 0.027 | 4.2 | 0.31 | 0.08 | 0.14 | 0.0168 |
| 8-DB | 27.6 | 8.0 | 0.09 | 0.0239 | 0.076 | 16.9 | 21.5 | 0.0121 | 2.4 | 0.144 | 0.06 | 0.06 | 0.0066 |
| 23-DB | 596 | 6.4 | 0.16 | 0.379 | 0.164 | 42.0 | 338.0 | 0.175 | 7.20 | 0.460 | 0.24 | 0.93 | 0.094 |
| 9-BB | 18.1 | 8.7 | 0.06 | 0.014 | 0.03 | 27 | 14.6 | 0.007 | 1.9 | 0.14 | 0.03 | 0.05 | 0.0045 |
| 10-BB | 30.1 | 11.7 | 0.10 | 0.025 | 0.061 | 37 | 23.9 | 0.014 | 3.8 | 0.23 | 0.04 | 0.09 | 0.0092 |
| 11-BB | 31.4 | 13.5 | 0.10 | 0.0236 | 0.088 | 29.1 | 24.9 | 0.0135 | 3.06 | 0.280 | 0.05 | 0.06 | 0.0112 |
| 12-CB | 63.9 | 14.1 | 0.07 | 0.0929 | 0.05 | 15.5 | 47.5 | 0.0410 | 4.76 | 0.188 | 0.11 | 0.10 | 0.0383 |
| 13-CB | 16.3 | 14.7 | 0.14 | 0.0159 | 0.052 | 25.5 | 13.512 | 0.0089 | 2.75 | 0.238 | 0.19 | 0.05 | 0.0053 |
| 14-EB | 19 | 21.5 | 0.12 | 0.014 | 0.031 | 30 | 13.1 | 0.006 | 2.1 | 0.21 | 0.04 | 0.09 | 0.0045 |
| 15-EB | 10 | 24.4 | 0.15 | 0.0082 | 0.095 | 23.8 | 9.99 | 0.0058 | 1.26 | 0.444 | 0.03 | 0.08 | 0.005 |
| 16-MS | 27.3 | 15.6 | 0.08 | 0.0280 | 0.055 | 17.6 | 24.6 | 0.0167 | 3.22 | 0.121 | 0.12 | 0.06 | 0.0116 |
| 17-MS | 35.8 | 15.5 | 0.08 | 0.026 | 0.019 | 20 | 25.7 | 0.012 | 2.6 | 0.19 | 0.04 | 0.07 | 0.0116 |
| 18-MS | 62.3 | 15.2 | 0.15 | 0.051 | 0.049 | 25.5 | 39.25 | 0.024 | 3.2 | 0.325 | 0.09 | 0.12 | 0.0128 |
| 19-WB | 71.1 | 14.7 | 0.10 | 0.0915 | 0.142 | 22.3 | 56.3 | 0.0421 | 4.56 | 0.628 | 0.08 | 0.17 | 0.0244 |
| 20-SB | 84.3 | 7.3 | 0.09 | 0.2759 | 0.084 | 11.8 | 63.9 | 0.1235 | 3.56 | 0.185 | 0.09 | 0.15 | 0.0376 |
| 21-SB | 137 | 17.4 | 0.12 | 0.12 | 0.123 | 37 | 95.3 | 0.052 | 4 | 0.46 | 0.08 | 0.27 | 0.0344 |
| 22-SB | 97.4 | 13.3 | 0.18 | 0.079 | 0.083 | 25.5 | 64.1 | 0.0375 | 3.65 | 0.245 | 0.12 | 0.18 | 0.0212 |
