# Supplementary material for: A benthic bioindicator reveals distinct land and ocean–Based influences in an urbanized coastal embayment
Source: PLoS One. 2018 Oct 11;13(10):e0205408. doi: 10.1371/journal.pone.0205408 (PMC6181360; doi:10.1371/journal.pone.0205408)
Supplement: S4 Table — Major and trace element values from Site 23-DB were not included in Z-score calculations due to contamination concerns. (DOCX) [file pone.0205408.s004.docx]

| **Site** | **Al** | **As** | **Cd** | **Ce** | **Co** | **Cu** | **Fe** | **La** | **Mn** | **Ni** | **Pb** | **V** | **Y** |
| --- | --- | --- | --- | --- | --- | --- | --- | --- | --- | --- | --- | --- | --- |
| 1-Off | -1.17 | 0.70 | 0.36 | -0.76 | 1.37 | -2.05 | -1.12 | -0.64 | -1.62 | -0.68 | -0.38 | -0.24 | -0.78 |
| 2-Off | -0.81 | -0.22 | 0.85 | -0.50 | -1.17 | -1.19 | -0.85 | -0.54 | -1.38 | -1.28 | -1.10 | -0.84 | -0.89 |
| 3-Off | -0.63 | 0.17 | 0.72 | -0.58 | -0.41 | -0.51 | -0.76 | -0.58 | -1.40 | 1.13 | -0.98 | 1.04 | -0.77 |
| 4-Off | 0.00 | 0.42 | 1.53 | -0.02 | -0.53 | -0.89 | -0.28 | -0.05 | -0.92 | 1.35 | -0.50 | 2.14 | -0.38 |
| 5-Off | -0.88 | -0.57 | 3.75 | -0.64 | -0.41 | -1.26 | -0.77 | -0.59 | -1.25 | -0.50 | -0.78 | 0.64 | -0.82 |
| 6-DB | 1.22 | -2.07 | -0.24 | 1.78 | 0.42 | 1.61 | 1.46 | 2.32 | 0.75 | -0.44 | 0.73 | 0.61 | 2.14 |
| 7-DB | 0.81 | -1.01 | -0.40 | 0.06 | 1.16 | 1.81 | 0.85 | -0.02 | 1.18 | 0.31 | 0.23 | 0.25 | 0.16 |
| 8-DB | -0.52 | -1.21 | -0.50 | -0.50 | 0.18 | -0.52 | -0.50 | -0.51 | -0.27 | -0.91 | -0.23 | -0.94 | -0.65 |
| 9-BB | -0.79 | -1.07 | -0.56 | -0.66 | -1.23 | 0.42 | -0.79 | -0.68 | -0.68 | -0.94 | -0.98 | -1.17 | -0.82 |
| 10-BB | -0.44 | -0.42 | -0.47 | -0.49 | -0.28 | 1.35 | -0.40 | -0.45 | 0.86 | -0.28 | -0.74 | -0.54 | -0.45 |
| 11-BB | -0.41 | -0.02 | -0.46 | -0.51 | 0.55 | 0.61 | -0.36 | -0.46 | 0.26 | 0.10 | -0.58 | -0.86 | -0.28 |
| 12-CB | 0.54 | 0.11 | -0.54 | 0.57 | -0.62 | -0.65 | 0.58 | 0.45 | 1.63 | -0.59 | 1.03 | -0.31 | 1.88 |
| 13-CB | -0.84 | 0.25 | -0.38 | -0.63 | -0.56 | 0.28 | -0.83 | -0.62 | 0.02 | -0.21 | 2.95 | -1.14 | -0.75 |
| 14-EB | -0.77 | 1.73 | -0.42 | -0.66 | -1.20 | 0.70 | -0.85 | -0.71 | -0.52 | -0.43 | -0.74 | -0.54 | -0.82 |
| 15-EB | -1.03 | 2.37 | -0.35 | -0.75 | 0.76 | 0.12 | -0.98 | -0.72 | -1.19 | 1.30 | -0.98 | -0.55 | -0.78 |
| 16-MS | -0.53 | 0.44 | -0.52 | -0.44 | -0.47 | -0.46 | -0.37 | -0.36 | 0.39 | -1.08 | 1.11 | -1.00 | -0.25 |
| 17-MS | -0.28 | 0.42 | -0.53 | -0.47 | -1.57 | -0.24 | -0.33 | -0.52 | -0.11 | -0.57 | -0.74 | -0.85 | -0.26 |
| 18-MS | 0.49 | 0.35 | -0.36 | -0.08 | -0.64 | 0.28 | 0.24 | -0.12 | 0.37 | 0.42 | 0.47 | -0.07 | -0.16 |
| 19-WB | 0.74 | 0.25 | -0.47 | 0.54 | 2.22 | -0.02 | 0.95 | 0.48 | 1.47 | 2.67 | 0.14 | 0.71 | 0.77 |
| 20-SB | 1.13 | -1.37 | -0.49 | 3.40 | 0.42 | -1.00 | 1.27 | 3.18 | 0.66 | -0.61 | 0.54 | 0.48 | 1.83 |
| 21-SB | 2.66 | 0.83 | -0.24 | 0.99 | 1.62 | 1.35 | 2.57 | 0.81 | 1.02 | 1.42 | 0.23 | 2.29 | 1.57 |
| 22-SB | 1.51 | -0.07 | -0.28 | 0.35 | 0.39 | 0.28 | 1.27 | 0.33 | 0.74 | -0.17 | 1.31 | 0.88 | 0.51 |

**S4 Table. Major and trace element Z-scores of M. plebejus muscle composites for each sample site in Moreton Bay**. Major and trace element values from Site 23-DB were not included in Z-score calculations due to contamination concerns
